# Biostimulation Shaped Microbial Communities in Oil-contaminated Desert Soils

**DOI:** 10.1007/s00284-026-04756-x

**Published:** 2026-02-10

**Authors:** Zheng Li, Mitiku Mihiret Seyoum, Ravid Rosenzweig, Faina Gelman, Zeev Ronen

**Affiliations:** 1 Zuckerberg Institute for Water Research, Jacob Blaustein Institutes for Desert Research, Ben-Gurion University of the Negev, 84990 Sede Boqer Campus, Be′er Sheva, Israel; 2 Geological Survey of Israel, 32 Yeshayahu Leibowitz St, Jerusalem, 9692100 Israel; 3 Department of Crop, Soil, and Environmental Sciences, University of Arkansas, Fayetteville, AR USA; 4 Present Address: Department of Environmental Sciences, University of California, Riverside, CA USA

## Abstract

Oil contamination poses serious environmental challenges, particularly in hyper-arid desert ecosystems; yet, microbial responses to oil pollution and biostimulation in desert soils remain insufficiently explored. This study explored the microbial community dynamics and hydrocarbon degradation potential to oil contamination and biostimulation in desert soils collected from sites in southern Israel polluted in 1975 and 2014. Laboratory-based biostimulation experiments were conducted over 1.5 years, involving the addition of water (20% or 50% gravimetric saturation), nutrients, and biosurfactants. Results revealed that biostimulation treatments reduced microbial diversity but enriched populations capable of hydrocarbon degradation. *Proteobacteria* and *Actinobacteria* were the dominant phyla, comprising 68% to 78% of the total microbial community across both contamination timelines. The relative abundance of *Chloroflexi* was higher in biostimulated contaminated soils, showing an increase of 41% to 227% compared to untreated contaminated soils. Differential abundance analysis identified distinct taxa of hydrocarbon degraders associated with untreated contaminated soils (e.g., *Pseudomonas*, *Alkanindiges*, *Bacillus*, and *Mycobacterium*) and biostimulated contaminated soils (e.g., *Pseudomonas*, *Flavobacterium*, *Pseudoxanthomonas*, unclassified *Microbacteriaceae*, *Solimonadaceae*, and *Gammaproteobacteria*). Further, soil hydrophobicity and total petroleum hydrocarbon suggested a positive relationship with the abundance of the *nahAc* gene, a key marker of hydrocarbon degradation. These findings exhibit that targeted biostimulation with water, nutrients, and biosurfactants accelerates oil biodegradation while selectively reshaping microbial communities toward hydrocarbon degrading taxa in hyper-arid desert soils. This study points to the potential development of effective management and remediation strategies for oil-contaminated desert environments.

**Supplementary Information:**

The online version contains supplementary material available at 10.1007/s00284-026-04756-x.

## Introduction

Oil is indispensable for energy production, transportation, and industrial processes; however, its extraction, transportation, and storage pose risks of environmental contamination. Oil spills result in severe damage to ecosystems, lasting for decades. Direct or indirect contact with crude oil can cause deleterious health risks to humans and damage different animal and plant species [1]. The Evrona Nature Reserve in southern Israel experienced two oil spills from pipeline leaks, the first in 1975 and the second 39 years later in December 2014 [2]. The two contaminated areas are situated near each other, with a few hundred meters distance between them [3]. It has been shown that oil pollution induced severe soil hydrophobicity [4, 5], reduced young tree density [3], inhibited seed germination and growth [2], and diminished bacterial diversity [6]. Therefore, the remediation of hydrocarbon contamination at this site is vital to this fragile and unique desert ecosystem. As this delicate ecosystem is sensitive to physical disturbance, in situ treatment with minimal disruption is needed.

Bioremediation approaches, such as bioaugmentation and biostimulation, are promising, cost-effective, and environmentally sustainable methods for addressing hydrocarbon contamination. Bioaugmentation involves the addition of specific degrading microorganisms to accelerate the rate of degradation. For instance, *Pseudomonas* strains isolated from Evrona contaminated soils have demonstrated efficacy in degrading long-chain hydrocarbons [7]. Biostimulation, on the other hand, enhances environmental conditions such as moisture, nutrients (nitrogen and phosphorous), oxygen, pH, salinity, and temperature to stimulate indigenous hydrocarbon-degrading microbial communities. The recommended carbon-to-nitrogen-to-phosphorus ratio (C: N: P) for effective hydrocarbon biodegradation range from 100:10:1 to 100:20:1 [8, 9]. In addition, the optimal water saturations for hydrocarbon removal range from 30% to 90%, where high saturations may limit oxygen availability and decrease microbial activity [10].

In hyper-arid desert soils, moisture and nutrient availability often constrains biodegradation potential. Previous studies investigated the hydrocarbon degraders in desert oil-polluted soil habitats. For instance, the high influx of carbon in nutrient-poor desert soils can significantly affect the soil microbial community selection for oil-degrading bacteria [11]. Indigenous microorganisms may be able to adapt to oil contamination, and the community structure can shift towards a hydrocarbon-degrading bacteria-dominating structure in desert soil saturated with oil [12]. For instance, *Actinobacteria* were the predominant hydrocarbon-utilizing species in Kuwait’s nutrient-poor desert soil samples [13]. In a study on desert soil contaminated by crude oil from Oman, the addition of sewage sludge and wheat straw stimulated the degradation of C14 to C30 alkanes, pointing to the presence of biodegradation potential even in dry soil [14]. Furthermore, nutrient supplementation has improved hydrocarbon degradation rates in other desert ecosystems, highlighting the effectiveness of biostimulation compared to bioaugmention alone [15].

A study conducted two months after the spill of 2014 revealed that *Proteobacteria* numbers increased dramatically in contaminated soils compared to clean soils [6]. Moreover, microbial community diversity was lower in the soils polluted in 1975 than in those polluted in 2014, indicating a persistent adverse impact of the contamination on the microbial populations. A combined treatment of mineral fertilization and maintaining 50% water saturation significantly accelerated the hydrocarbon degradation in the sediments from the Evrona hyper-arid region [16]. While biostimulation has been studied in various environments, its long-term effects on microbial communities in desert soils with different pollution histories remain poorly understood. The current study aimed to fill this gap by examining how biostimulation through the addition of water, nutrients, and biosurfactants affects microbial community composition and hydrocarbon degradation potential in soils contaminated by two distinct oil spills. Specifically, our objectives were to (1) characterize microbial community dynamics in response to biostimulation treatments, (2) elucidate the relationships between microbial taxa, functional hydrocarbon degradation genes, and soil physicochemical properties, and (3) predict the identity of potential hydrocarbon-degrading microorganisms associated with these functional genes. Our research provides novel insights into effective bioremediation practices and the critical interplay between microbial populations and oil-induced soil changes in desert environments.

## Materials and Methods

### Soil Sampling and Incubation

The soil samples utilized in this study were initially collected from two oil spill sites contaminated in 1975 and 2014, located at Evrona Nature Reserve in southern Israel (Fig. S1). Clean soils were collected from the same geographical region as natural controls. Soil samples (0–7 cm depth) collected in late May 2017 were analyzed for physical and chemical properties at the ISO-17,025 certified Neve Yaar Service Laboratory. The analyses included measurements of pH, electrical conductivity (EC), Olsen phosphorus (P), and ammonium nitrogen (N-NH₄). The pH values of soils polluted in 1975 and 2014 ranged from 7.0 to 7.8, whereas the clean soil exhibited a higher pH (> 8.1). Electrical conductivity in the 2014 polluted soils was below 1 dS/m, compared to 3.5 dS/m in the clean soil. In the 1975 samples, EC values were below 1 dS/m in contaminated soils. P levels were below the laboratory detection limit of 3.0 mg/kg in all samples. In contrast, N-NH₄ concentrations ranged from 5 to 15 mg/kg in the polluted soils but remained below 5 mg/kg in the clean soils. The total organic matter in the soil was measured “loss-on‐ignition” method. The concentration of TOC (natural and oil-contaminated) was 6.2 ± 0.097% for the 2014 contaminated soil, 1.94 ± 0.166% for the 1975 contaminated soil, and 0.9 ± 0.256% for the clean soil.

Following protocols from a previous incubation experiment [5], soils were incubated in laboratory conditions beginning in late 2017 for 1.5 years. As summarized in Table 1, seven treatments without replications (single microcosm) were established for the 2014 site soils, including 14clean, 14control, 14W20, 14W20Nut, 14W20Surf, 14W20NutSurf, and 14W50. Three treatments were applied for the 1975 site soils, including 75control, 75W20, and 75W20Nut. During the incubation, soil batches (about 2 kg each in a single container) were incubated at 25 °C and mixed every two or three days to maintain sufficient oxygen levels within the sealed containers. Soil hydrophobicity and hydrocarbon concentrations were monitored throughout the experimental period, with full methods and data previously published [5]. In brief, 30 g soil samples were collected at regular intervals to determine the hydrophobicity in terms of their water drop penetration time (WDPT) [17] and molarity of ethanol droplet (MED) [18] techniques. In addition, 10 g soil samples were collected at the same time intervals for the total petroleum hydrocarbon (TPH) analysis using gas chromatography-mass spectrometry (GC-MS). The GCMS analysis methodology and TPH measurements were described before (Zheng et al. 2021 [5]). (Fig. S2). In this analysis, we quantified the alkane fraction from C14 to C34. We examine the relative abundances of specific hydrocarbons such as Pr/n-C17 and Ph/n-C18. Chromatograms of oil from the control and treated soil, before and after incubation, with clearly labeled alkane peaks are shown in Supplementary Figure S2. At the end of incubation, soil samples were collected and stored at -20 °C until further DNA extraction.

**Table 1:** Laboratory incubation treatments for soils collected from 2014 and 1975 sites

Abbreviation	Soil origin	Sampling time	Treatment
14cleanZero	2014 site	Start of incubation	Clean soil
14controlZero	2014 site	Start of incubation	Untreated contaminated soil
14clean	2014 site	End of incubation	Clean soil
14control	2014 site	End of incubation	Untreated contaminated soil
14W20	2014 site	End of incubation	20% water saturation^a^
14W20Nut	2014 site	End of incubation	20% water saturation with the addition of nutrients^a,b^
14W20Surf	2014 site	End of incubation	20% water saturation with the addition of biosurfactants^a,c^
14W20NutSurf	2014 site	End of incubation	20% water saturation with the addition of nutrients and biosurfactants^a,b,c^
14W50	2014 site	End of incubation	50% water saturation^a^
75controlZero	1975 site	Start of incubation	Untreated contaminated soil
75control	1975 site	End of incubation	Untreated contaminated soil
75W20	1975 site	End of incubation	20% water saturation^a^
75W20Nut	1975 site	End of incubation	20% water saturation with the addition of nutrients^a,b^

a: Water saturation was calculated based on field measurements of a bulk density of 1.5 g cm^− 3^, corresponding to a calculated porosity of 43%.

b: The nutrient solution consisted of 3 g L^− 1^ ammonium sulfate and 150 mg L^− 1^ potassium phosphate dissolved in tap water (deionized water was not used to avoid soil dispersion).

c: Rhamnolipids (Agaetech, USA) at 200 mg L^− 1^ were used to test the effect of surfactants on oil degradation and hydrophobicity reclamation.

## DNA Extraction, Real-Time Polymerase Chain Reaction (qPCR), and 16S rRNA Amplicon Sequencing

Microbial DNA was extracted from 0.25 g dry weight (dw) soils using the DNeasy PowerLyzer PowerSoil kit (Qiagen, Hilden, Germany) at the start and end of incubation. Two independent extractions were performed on each of the soil samples, and the DNA from each was pooled together. The quantity and quality of DNA were estimated by NanoDrop ND-1000 (Thermo Fisher Scientific Wilmington, DE). A280/260 of between 1.8 and 2 and A260/A230 of between 1.5 and 2 was considered acceptable for amplification. Extracted DNA was used to quantify *alkB*,* nahAc*, and *phe* genes using degenerated primers listed in Supplementary Table 1. Each qPCR reaction (20 µL total volume) consisted of of 10 µL qPCR-BIO SyGreen Blue Mix Lo-Box (PCR Biosystems Inc, USA), 0.4 µM forward primer, 0.4 µM reverse primer, 1 µL DNA template (less than 20 ng), and 8.2 µL nuclease-free water. Amplifications were performed using CFX96 Touch™ Real-Time PCR Detection Systems (Bio-Rad, Hercules, CA, USA), with an initial denaturation at 94 °C for 30 s, followed by 40 cycles (94 °C for 5 s, 64 °C for 15 s, 72 °C for 10 s) and a final hold at 55 °C for 30 s. PCR products were checked for specificity using 1.5% agarose gel electrophoresis. Purified PCR products were cloned into a pJET1.2/blunt vector according to the manufacturer’s instructions (Thermo Fisher Scientific cloneJET, K-1231), transferred into competent cells (*Escherichia coli* JM109), and cultured on LB-amp plates (ampicillin 100 µg. mL − 1) at 37 °C. Plasmids were purified using the Bioneer AccuPrep K-3030 plasmid extraction kit and sequenced at the National Institute for Biotechnology (Ben Gurion University of the Negev, Israel). Sequences were manually scanned for matches using the BLASTn algorithm with the respective gene.

Additionally, DNA extracts were used to amplify the V3-V4 region of the 16 S rRNA gene (primer details in Supplementary Table 1). Amplicon sequencing was conducted at the University of Illinois at Chicago Core for Research Informatics (UICCRI) using the Illumina MiSeq platform with CS1-341 F-CS2-806R primers [19].

The raw sequences were submitted to the National Center for Biotechnology Information (NCBI) under the bio project PRJNA1188154 (accession numbers SAMN44826220 to SAMN44826245).

## Microbial Community Analysis

The forward and reverse reads in fastq format were processed using Mothur [20]. The raw sequences were trimmed and aligned for the SILVA bacteria database (Release 138), and classified into operational taxonomic units (OTU) at a 0.03 cutoff after removing chimeras and non-bacterial sequences. The taxonomy classification of the OTUs and the downstream analysis were conducted in RStudio (version 1.0.5042) [21]. The R packages file2meco (version 0.2.0) [22], phyloseq (version 1.36.0) [23], and microbiome (version 1.14.0) [24] were used to generate bar plots at the phylum and class levels and Venn plots of the OTU abundance. Alpha diversity indices (Chao1, ACE, Shannon, Simpson, Inverse Simpson, Fisher) and principal coordinate analysis (PCoA) were conducted using the packages microbiome (version 1.14.0) [24] and phyloseq (version 1.36.0) [23]. Heat maps illustrating the most abundant OTUs were generated using packages ampvis2 (version 2.7.11) [25], microbiome (version 1.14.0) [24], and phyloseq (version 1.36.0) [23]. The differentially abundant phylotypes between the treatments were determined by the Wald test coupled with a parametric model using the R packages DESeq2 for differences exploration [26] and microbiome (version 1.26.0) [24].

## Function Prediction by PICRUSt2

PICRUSt2 [27] was used to predict the microbial functions of the sequencing data from the Kyoto Encyclopedia of Genes and Genomes (KEGG) orthologs (KO) [28]. Analyzed functional genes and their KEGG numbers are detailed in Supplementary Table 2. The connections between the phylotypes and genes associated with hydrocarbon degradation were created with the circlize package (version 0.4.13) [29]. Genera with the most abundant relative abundance for each set of functional genes were selected for building the chord diagrams (taxon_rel_function_abun > 0.005).

## Statistics Analysis

Since the experimental treatment consisted of a single microcosm and no independent biological replicates were included in the incubation experiment, the dataset does not support formal hypothesis testing or statistically generalizable inference. Multivariate analyses, differential abundance analyses, and correlation analyses were therefore conducted for exploratory purposes, aiming to identify patterns, trends, and associations that can help generate testable hypotheses for future studies. Correlations between the abundance of functional genes quantified by qPCR and soil variables, as well as the correlations between predicted functions and the top 20 abundant genera, were assessed using Spearman’s rank correlation. These correlations were used to explore associations between gene abundance, predicted functions, and soil variables. P-values are reported as descriptive metrics. Soil variables included water drop penetration time (WDPT), molarity of ethanol droplet (MED), and total petroleum hydrocarbons (TPH) as reported in a previous study [5].

## Results

### Microbial Community Structure

**Fig. 1:**
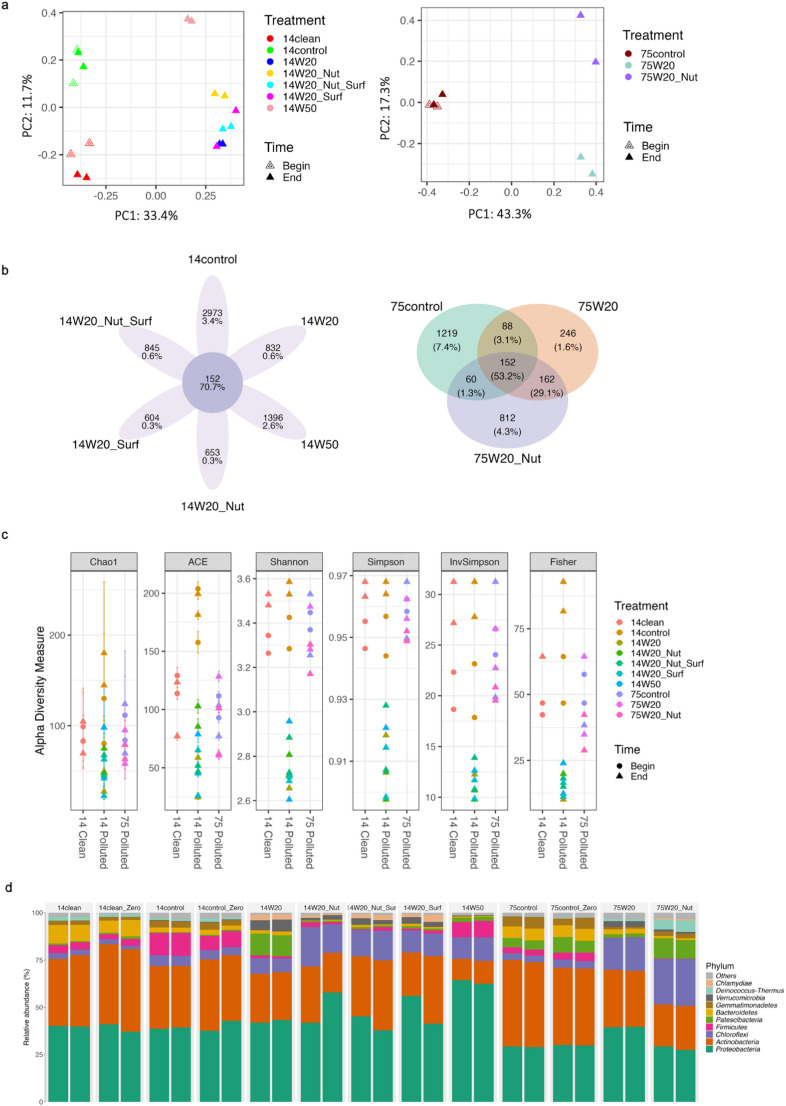
Principal Coordinates Analysis (PCoA) plots of microbial communities in soil samples collected in 2014 and 1975 (**a**). Venn diagrams showing the number and percentage of shared and unique OTUs among treatments in the 2014 and 1975 soils (**b**). Alpha diversity indices (Chao1, ACE, Shannon, Simpson, Inverse Simpson, Fisher) for the 2014 and 1975 soils (**c**). Different treatments are indicated by color, and sampling times (beginning and end of incubation) are distinguished by symbol shape (circle = begin, triangle = end). Relative abundance of the most abundant bacterial phyla in soil samples collected in 2014 and 1975 under different treatment conditions (**d**)

Distinct microbial community structures were observed among treatments for soils contaminated in 1975 and 2014 (Fig. 1a). PCoA revealed four different clusters of microbial communities in the 2014 soils. The first cluster included untreated contaminated soils (14control and 14control_Zero), while the second comprised uncontaminated soils (14clean and 14clean_Zero). The third cluster contained soils amended with 20% water (14W20, 14W20Nut, 14W20Surf, and 14W20NutSurf), and the fourth included soils with 50% water content (14W50). Among 2014 soils, the highest proportion of unique bacterial OTUs were found in 14control (3.4%), followed by 14W50 (2.6%), with fewer unique OTUs detected in other treatments (Fig. 1b). In the 1975 soils, the most unique OTUs appeared in 75control. Additionally, 75W20 and 75W20Nut shared 29.1% of their OTUs, illustrating notable overlap in these treatments. Richness (Chao1, ACE) and diversity indices (Shannon, Simpson, Inverse Simpson, Fisher) indicated higher diversity in untreated 2014 soils compared to biostimulated soils, whereas no consistent trend emerged for the 1975 soils (Fig. 1c). As shown in Fig. 1d, *Actinobacteria* and *Proteobacteria* accounted for approximately 68% to 78% of the bacterial populations in all the 1975 and 2014 soil treatments. At the end of incubation, *Chloroflexi* were more abundant in the 14W20 (7.77%), 14W20Nut (17.97%), 14W20Surf (11.64%), 14W20NutSurf (14.60%), and 14W50 (11.82%) compared to the 14control soils (5.49%). In contrast, the abundance of *Firmicutes* was decreased in the 14W20 (1.34%), 14W20Nut (1.84%), 14W20Surf (1.33%), and 14W20NutSurf (1.27%) compared to the 14control soils (12.13%) but was still enriched in the 14W50 (8.33%). No notable changes were observed in the microbial communities of 14control and 14clean soils from the beginning to the end of incubation. Similarly, in the 1975 soils, *Chloroflexi* increased in 75W20 and 75W20Nut compared to 75control, while the abundance of *Firmicutes* decreased in these treatments compared to the controls.

The top 30 abundant OTUs in 2014 and 1975 soils are shown in Fig.S3. By the end of incubation, the top five enriched OTUs in the 2014 biostimulated treatments (14W20, 14W20Nut, 14W20NutSurf, 14W20Surf and 14W50) were associated with the phyla *Chloroflexi*,* Proteobacteria*,* and Actinobacteria* (Fig.S3a). Notably, *Pseudomonas* OTU was higher in 14W50 compared to all other 2014 treatments. Similarly, several OTUs from *Actinobacteria*,* Proteobacteria*, and *Chloroflexi* were more abundant in 75W20 and 75W20Nut than in 75control at the end of incubation (Fig.S3b). Interestingly, *JG3-KF-CM45_ge*, a member of the *Chloroflexi* phylum, exhibited enrichment in both 2014 and 1975 biostimulation treatments.

## Differentially Abundant Phylotypes

**Fig. 2:**
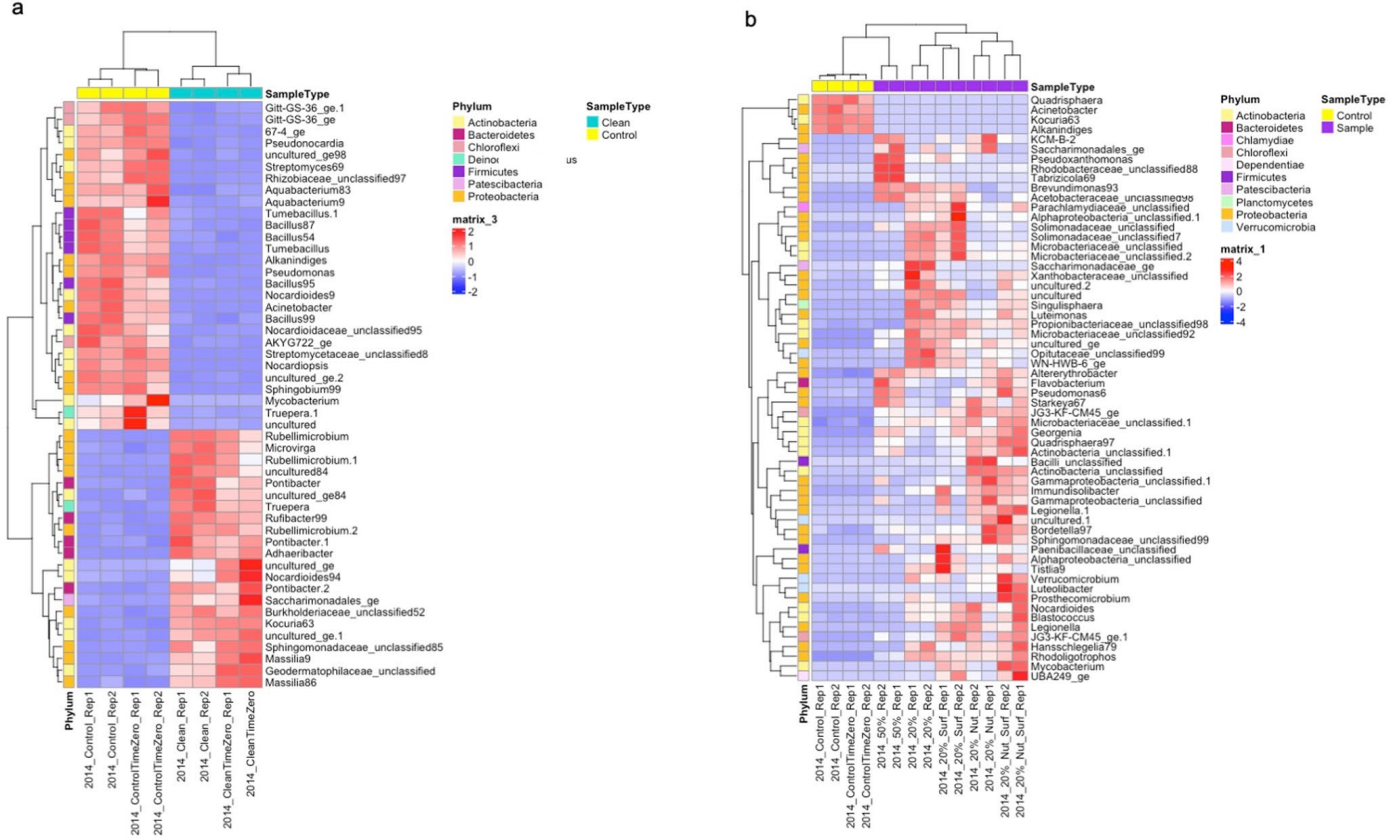
Heatmap of the top 50 differentially abundant taxa between the 2014 clean and control soil samples (**a**) as well as between the 2014 control soils and other 2014 soil treatments (**b**), as determined by DESeq analysis. Taxa are clustered by abundance profiles, with sample types (clean, control) and bacterial phyla indicated by color annotations. The heatmap color scale represents normalized abundance (red = higher, blue = lower). Statistical analyses are exploratory due to the absence of biological replication.

The DESeq analysis was conducted to further investigate differential abundances between treatments, and to determine differentiated phylotypes in the 2014 soils (Fig. 2) and 1975 soils (Fig.S4). Several phylotypes showed higher relative abundance in specific treatments, indicating contrasting taxonomic patterns across experimental conditions. *Pseudomonas*, *Alkanindiges*, *Bacillus*, *Mycobacterium* and *Acinebacter* enriched in the 14control soils (Fig. 2a). When comparing 14control to the other biostimulated treatments, specific phylotypes enriched in 14W50 included *Pseudomonas*, *Flavobacterium*, *Pseudoxanthomonas*, and *Tabrizicola*. Additionally, unclassified *Microbacteriaceae*, unclassified *Solimonadaceae*, unclassified *Propionibacteriaceae*, and *Singulisphaera* were also enriched in 14W20 and 14W20Surf (Fig. 2b). For 14W20Nut and 14W20NutSurf treatments, the enriched phylotypes included *Georgenia*, *Immundisolibacter*, *Legionella* and unclassified *Gammaproteobacteria*. The abundance of *Pseudomonas* also showed substantial enrichment in 14W20NutSurf.

In the 1975 soils, a group of phylotypes were commonly enriched in both 75W20 and 75W20Nut, including unclassified *Gemmatimonadetes*, *JG3-KF-CM45_ge*, *JG3-KF-CM66_ge*, and *Hansschlegelia* (Fig.S4). When comparing 14control and 75control, enriched phylotypes in 14control included *Pseudomonas*, *Pseudonocardia*, *Bacillus*, and *Mycobacterium* in the 14control (Fig.S5).

### Functions and Correlations

**Fig. 3:**
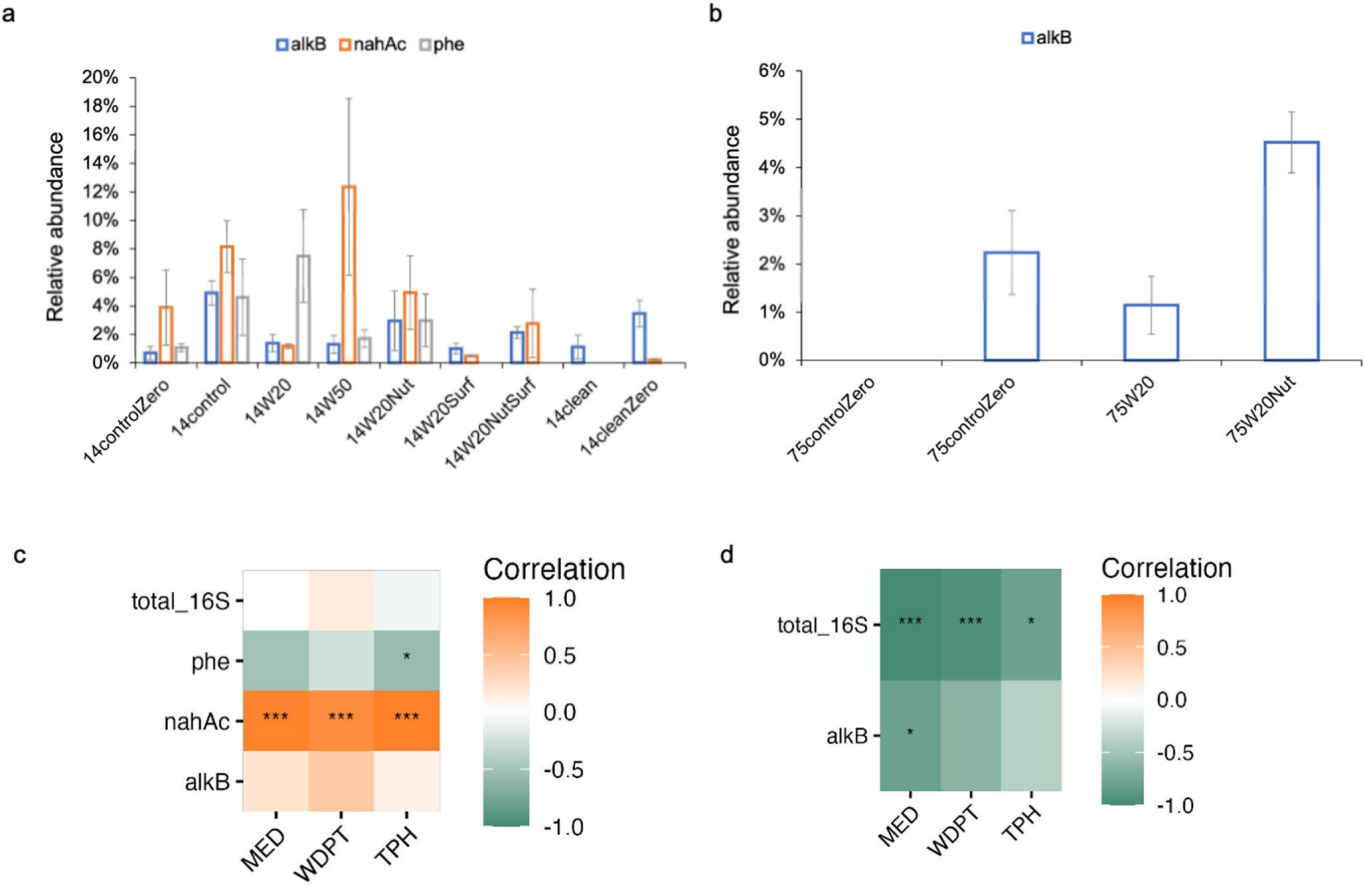
The relative abundance of alkane monooxygenase gene *alkB*, naphthalene dioxygenase *nahAc*, and phenol monooxygenase gene *phe* in 2014 (**a**) and 1975 (**b**) soil samples; Heatmap of the correlations between the abundance of genes (copies/dry weight of soil) and soil properties (WDPT, MED, and TPH) in the 2014 contaminated (**c**) and 1975 contaminated (**d**) samples. “*” indicates *p* < 0.05, “**” indicates *p* < 0.01 and “***” indicates *p* < 0.001. Statistical analyses are exploratory due to the absence of biological replication.

The ratios of abundances of hydrocarbon degradation-related genes (*alkB*, *nahAc*, and *phe*) to 16 S rRNA genes are shown in Fig. 3a and b. Notably, the *nahAc* gene showed the highest relative abundance in 14W50 (12.35%), followed by 14control (8.16%) and 14W20Nut (4.94%). The gene *phe* was most enriched in 14W20Nut (7.51%), followed by 14W50 (4.61%), and then in 14W20NutSurf (2.69%). Additionally, 14control samples exhibited a high abundance of the *alkB* gene (4.91%).

**Fig. 4:**
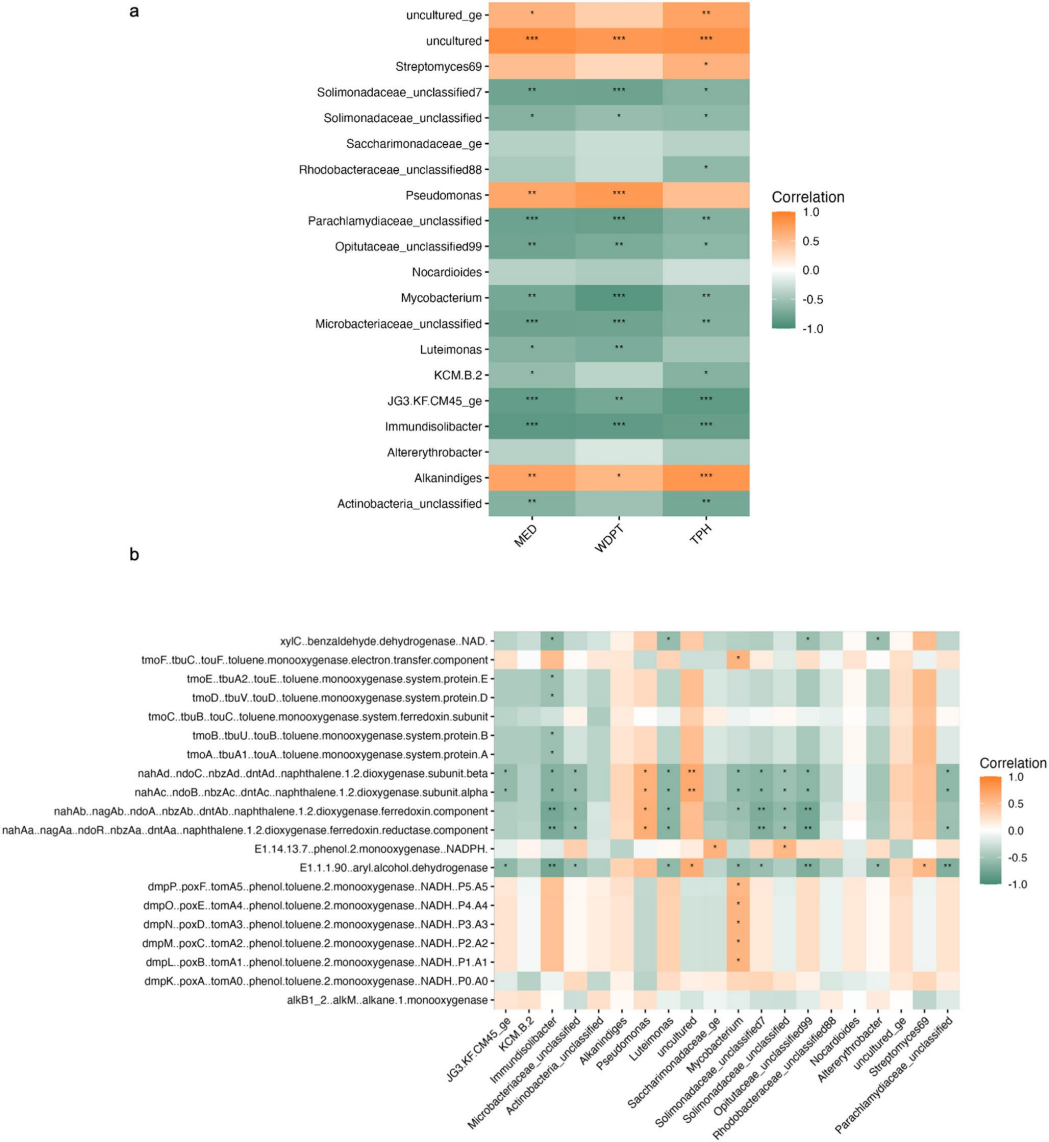
Heatmap of Spearman correlations between the relative abundance of top 20 bacterial genera and soil properties in the 2014 contaminated soils. Soil properties include water drop penetration time (WDPT), molarity of ethanol droplet (MED), and total petroleum hydrocarbons (TPH) (**a**). Heatmap showing the correlations between the abundance of top 20 genera and predicted functional pathways, as determined by PICRUSt2 analysis, in the 2014 contaminated soils (**b**). The color scale indicates correlation coefficients (orange = positive correlation, green = negative correlation). “*” indicates *p* < 0.05, “**” indicates *p* < 0.01 and “***” indicates *p* < 0.001. Statistical analyses are exploratory due to the absence of biological replication.

Correlations between soil hydrophobicity, hydrocarbon contents, and gene abundance are presented in Fig. 3c and d. Mean values of WDPT, MED and TPH were summarized in Table S3. The abundance of the nahAc gene showed a positive association with WDPT, MED, and TPH, suggesting a potential link between aromatic hydrocarbon persistence and functional gene prevalence under the studied conditions (Fig. 3c). In the 1975 contaminated soil samples, the *alkB* gene, encoding alkane monooxygenase, negative trend values of WDPT, MED, and TPH (Fig. 3d). In contrast, a positive trend observed in 2014 contaminated soils (Fig. 3c). The correlations between soil hydrophobicity (as indicated by WDPT and MED) and hydrocarbon contents (measured by TPH) (Table S3) and the top 20 genera in contaminated samples (Table S4& S5) were analyzed for 2014 soils and 1975 soils (Fig. 4a and Fig.S6). In the 2014 soils, *Pseudomonas* and *Alkanindiges* showed positive correlations with both soil hydrophobicity and hydrocarbon contents (Fig. 4a). In addition, *Potibacter* and *Kocuria* were also correlated with these factors in the 1975 soils (Fig.S6). However, we emphasize that these correlations do not imply causality as no temporal analysis was conducted.

PICRUSt2 analysis predicted hydrocarbon degradation functional gene profiles based on the taxonomic data. In 2014, soil treatments, *Pseudomonas* correlated positively with genes encoding naphthalene dioxygenase, while *Mycobacterium* correlated with phenol/toluene monooxygenase. (Fig. 4b). In the 1975 soil treatment, *Blastococcus*, *Marmoricola*, and *Dietzia* showed positive correlations with subunits of naphthalene dioxygenase (Fig.S7). Phylotypes predicted to be associated with a subset of genes are shown using chord diagrams (Figures S8-S11). All four subunits of naphthalene dioxygenase were associated with *Pseudomonas* across all 2014 and 1975 soil treatments (Fig.S8 & S9). Additionally, as shown in Figs S10 & S11, six subunits of phenol/toluene monooxygenase were associated with a group of phylotypes in both the 2014 and 1975 soil samples. While these correlations may suggest potential functional shifts in the microbial communities, it is important to note that PICRUSt2 predictions are based on reference genome annotations and are inherently limited for low-abundance or unclassified OTUs.

## Discussion

Oil-contaminated desert soils pose a significant remediation challenge due to extreme environmental conditions, including high temperatures, limited water availability, and low organic matter content. Enhancing soil moisture and introducing essential nutrients can support bacterial growth and metabolic activity, particularly in nutrient-deficient desert environments. In this study, the amendment of oil-contaminated desert soils with water, nutrients, and biosurfactants effectively accelerated the degradation of hydrocarbons. These treatments also induced notable shifts in bacterial community composition, indicating the enrichment of hydrocarbon-degrading microbial populations, which could improve the bioremediation potential of these arid soils.

A potential limitation of this study is the lack of independent biological replication, as each treatment was represented by a single microcosm. This lowers the strength of inferential statistical analyses and limits the ability to draw generalizable conclusions. Accordingly, the results should be viewed as exploratory and descriptive. Nevertheless, the long-term incubation and integrative analytical approach provide valuable insights that can help guide the design of future replicated experiments and field-scale studies.

Microbial diversity decreased following biostimulation, suggesting selective enrichment of specialized hydrocarbon degraders. This pattern aligns with previous findings where diversity indices such as Shannon and ACE were notably higher in petroleum-contaminated soils compared to the same soils after 15% moisture remediation for 12 weeks, indicating lower microbial diversity post-remediation [30]. Similarly, in another study, diversity indices (Shannon, ACE, and Chao1) decreased during biostimulation with nitrogen and phosphorus compared to untreated contaminated soil [31], highlighting the role of nutrient enrichment in shaping microbial communities. A study on desert soils from Omen found that OTU richness increased with increasing hydrocarbon concentrations [32], suggesting that in arid, nutrient-limited environments, diverse microbial niches persist due to limited resource availability. In agreement with this, unique OTUs were mainly in the untreated oil-polluted soils with higher TPH (75control and 14control) compared to the other biostimulation treatments in our study. This observation suggests that hydrocarbon removal was not dependent on the overall microbial community diversity or richness. Instead, the microbial populations promoted in the treated contaminated soils may contribute more significantly to hydrocarbon degradation than other populations. We assumed that the observed decline in microbial diversity reflects a two-phase ecological process during biostimulation. In the early stage, the addition of water and nutrients likely favored fast-growing hydrocarbon degraders, leading to competitive exclusion of less-adapted taxa. Over the 1.5-year incubation period, the microbial community may have undergone niche specialization, resulting in a less diverse but functionally optimized consortium for hydrocarbon degradation. However, due to the lack of intermediate sampling points, we could not prove the temporal progression of these processes. To better understand the dynamics of microbial succession, future studies will include additional time-point sampling throughout the incubation period.

In the current study, *Proteobacteria* and *Actinobacteria* were the dominant phyla across all the soil treatments. Earlier studies reported that members of *Proteobacteria* (e.g., *Alpa-*,* Beta-* and *Gammaproteobacteria*) contain species that can degrade aliphatic and aromatic hydrocarbons [33]. Similarly, *Actinobacteria* are known to be potentially involved in the degradation of petroleum aromatic hydrocarbons (PAHs) [34]. Although *Proteobacteria* and *Actinobacteria* were predominant phyla in all the soil samples, specific taxa within these phyla varied among different biostimulated soil treatments. *Pseudomonas*, a genus within *Proteobacteria*, is widely recognized as a hydrocarbon degrader and many *Pseudomonas* isolates are capable of degrading diesel, crude oil, *n*-alkanes and PAHs [15, 35].

OTU affiliated with the genus *Pseudomonas* was increased in 14W50 than the other treatments at the end of incubation. In the 14W50 treatment, TPH decreased by 38.2% between the 12th and 18th months of incubation [5]. The results suggest that *Pseudomonas* may have been a functioning degrader during incubation. Indeed, we successfully isolated *Pseudomonas* strains producing biosurfactants from the 14W20NutSurf treatment [7]. According to our prior research on the removal of the oil during the experiment, TPH removal occurred rapidly during the initial months in the treatments 14W20, 14W20Nut, 14W20Surf, and 14W20NutSurf treatments, while for the 14W50 treatment, it lagged for one year before accelerating [5]. This pattern suggests that hydrocarbon degraders in 14W50 were either dormant or inhibited initially, only becoming active later in the incubation period. Further supporting this possibility is the high abundance of *nahAc* and *phe* genes observed in 14W50 at the end of the incubation phase. Additionally, the positive correlation trend between *Pseudomonas* abundance and both TPH levels and predicted naphthalene degradation functions further suggests that *Pseudomonas* can be involved in hydrocarbon degradation in the current study. Overall, *Pseudomonas* may play an important role in the hydrocarbon removal in Evrona desert soils.

OTU affiliated with the genus *Alkanindige* was increased in the 14control soils compared to 14clean soils. *Alkanindiges* genus, within the phylum *Proteobacteria*, has been widely reported as an alkane degrader in the past years [36, 37]. In particular, *Alkanindiges hydrocarboniclasticus* H1 was isolated from oil-contaminated sandy desert soil [37]. *Alkanindiges* has been identified as one of the most abundant genera in moderately hydrocarbon-polluted sites located in hot, arid climates, such as Kuwait [38].

OTU affiliated with the family *Microbacteriaceae*, within the phylum *Actionbacteria*, was also developed in 14W20 and 14W20Surf treatments. Members of *Microbacteriaceae* have been previously shown to thrive in motor oil-contaminated boreal soil [39]. The phylum has also been identified as PAH degraders in water and sediment environments [40]. Additionally, *Microbacterium deserti* strain SA5, a member of *Microbacteriaceae*, was recently isolated from hydrocarbon-contaminated soil [41]. Members of this phylum are also known to play a role in carbon and nitrogen cycling [42]. *Chloroflexi* showed greater abundance in biostimulated soil treatments compared to control and clean soils, indicating its potential role in biodegradation. Similarly, higher levels of *Chloroflexi* were observed in oil-contaminated soils compared to adjacent clean soils [43]. Additionally, another study observed an increase in *Chloroflexi* populations following the biotreatment of nutrients (C: N:*P* = 50:1:1) in oil-contaminated soil from an arid environment [44]. These trends support that *Chloroflexi* could be related to hydrocarbon removal in oil-contaminated soils. *Chloroflexi* has also been reported to be related to hydrocarbon metabolism in aged oil sludge-contaminated soils in Daqing and Shengli oilfields in China [45] and in crude oil-contaminated soils from the Daqing oilfield in another study [43].

The *nahAc* gene was negatively correlated with the concentrations of naphthalene and total PAHs [46], contrasting with our 2014 soils. Typically, crude oil-degrading bacteria communities follow a succession pattern: specialist alkane degraders evolved first (days after the spill), followed by specialist aromatic and polyaromatic degraders (months after the spill) [47]. In the current Evrona desert soils, the abundance of the *nahAc* gene (identified by qPCR) was positively correlated with soil hydrophobicity and TPH for the 2014 contaminated soils at the end of the incubation. Similarly, a positive correlation was found between nahAc and naphthalene concentrations in coal tar-contaminated soil sediments [48]. At low naphthalene concentrations, bacteria carrying the *nahAc* gene may be inactive [48]. Our previous research found that the TPH concentrations were approximately three to thirty times higher in the 2014 soils than in the 1975 soils at the end of incubation [5].

## Conclusions

This study provides an exploratory assessment of how long-term biostimulation with water, nutrients, and biosurfactants is associated with changes in microbial community composition and functional gene patterns in oil-contaminated desert soils. Although overall microbial diversity declined following biostimulation, several bacterial taxa known for hydrocarbon degradation were consistently enriched across treatments. Our study suggests that the addition of water, nutrients, and biosurfactants effectively promotes hydrocarbon degradation in desert soils contaminated by oil. Despite a reduction in overall microbial diversity, enrichment of hydrocarbon-degrading microbial taxa was evident. *Proteobacteria*, *Actinobacteria*, and *Chloroflexi* were identified as key phyla for hydrocarbon degradation in the biostimulated soils. Differential abundance analysis identified *Pseudomonas*, *Alkanindiges*, *Bacillus*, and *Mycobacterium* as the potential hydrocarbon degraders in the untreated 2014 soils, while *Pseudomonas*, *Flavobacterium*, *Pseudoxanthomonas*, unclassified *Microbacteriaceae*, unclassified *Solimonadaceae*, and unclassified *Gammaproteobacteria* were predominant in the biostimulated soils. *Pseudomonas* were likely to be the possible hydrocarbon degraders in the 2014 soil with 50% water saturation at the late stage of incubation. Notably, *Pseudomonas* showed a positive correlation with both TPH levels and predicted naphthalene degradation functions, further supporting its active role in hydrocarbon degradation. Additionally, the *nahAc* gene, a key marker for naphthalene degradation, was positively correlated with soil hydrophobicity and TPH concentrations in the 2014 soils, highlighting the persistence of PAHs at the end of the incubation period. These findings underline the effectiveness of biostimulation in enriching hydrocarbon-degrading microbial populations and enhancing the functional potential for hydrocarbon degradation in arid, oil-contaminated desert soils.

## Supplementary Information

Below is the link to the electronic supplementary material.

Supplementary Material 1

Supplementary Material 2

Supplementary Material 3

Supplementary Material 4

Supplementary Material 5

Supplementary Material 6

Supplementary Material 7

Supplementary Material 8

Supplementary Material 9

Supplementary Material 10

Supplementary Material 11

Supplementary Material 12

Supplementary Material 13

## Data Availability

The 16 S rRNA gene sequences were submitted to NCBI under the bio project PRJNA1188154 (accession numbers SAMN44826220 to SAMN44826245).
