# Supplementary material for: Biostimulation Shaped Microbial Communities in Oil-contaminated Desert Soils
Source: Curr Microbiol. 2026 Feb 10;83(4):167. doi: 10.1007/s00284-026-04756-x (PMC12891259; doi:10.1007/s00284-026-04756-x)
Supplement: Supplementary file 1 — Supplementary Material 1 [file 284_2026_4756_MOESM1_ESM.docx]

Supporting Information for

**Biostimulation Shaped Microbial Communities in Oil-contaminated Desert Soils**

Zheng Li^1,2*, a^, Mitiku Mihiret Seyoum^3^, Ravid Rosenzweig^2^, Faina Gelman^2^, Zeev Ronen^1*^

1 Zuckerberg Institute for Water Research, Jacob Blaustein Institutes for Desert Research, Ben-Gurion University of the Negev, 84990 Sede Boqer Campus, Israel

2 Geological Survey of Israel, 32 Yeshayahu Leibowitz St., 9692100 Jerusalem, Israel

3 Department of Crop, Soil, and Environmental Sciences, University of Arkansas, Fayetteville, AR, USA

a- present address: Department of Environmental Sciences, University of California, Riverside, CA, USA

* Correspondence:

[zli@ucr.edu](mailto:zli@ucr.edu)

[zeevrone@bgu.ac.il](mailto:zeevrone@bgu.ac.il)

Table S1. The sequences of the target hydrocarbon degradation genes.

| Primer | Target gene | Sequence | References |
| --- | --- | --- | --- |
| Alk-F | alkane monooxygenase (*alkB)* | 5’-GGTACGGSCAYTTCTACRTCGA-3’ | (Marchant et al. 2006) |
| Alk-R |  | 5’-CGGRTTCGCGTGRTGRT-3’ |  |
| NAH-F | Naphthalene dioxygenase (*nahAc*) | 5’-CAAAARCACCTGATTYATGG-3’ | (Baldwin et al. 2003) |
| NAH-R |  | 5’-AYRCGRGSGACTTCTTTCAA-3’ |  |
| PHE-F | Phenol monooxygenase  (*phe*) | 5’-GTGCTGACSAAYCTGYTGTT-3’ |  |
| PHE-R |  | 5’-CGCCAGAACCAYTTRTC-3’ |  |
| 341F | Bacteria 16S rRNA gene (V3-V4 region) | 5'-CCTACGGGAGGCAGCAG-3' | (Caporaso et al. 2011) |
| R806 |  | 5'-GGACTACHVGGGTWTCTAAT-3' |  |

Table S2. KO numbers and gene descriptions associated with hydrocarbon degradation.

| Gene name | Gene description |
| --- | --- |
| K00496 | *alkB1_2, alkM*; alkane 1-monooxygenase [EC:1.14.15.3] |
| K20938 | *ladA*; long-chain alkane monooxygenase [EC:1.14.14.28] |
| K03400 | *luxC*; long-chain-fatty-acyl-CoA reductase [EC:1.2.1.50] |
| K15853 | *luxD*; acyl transferase [EC:2.3.1.-] |
| K00494 | *luxA*; alkanal monooxygenase alpha chain [EC:1.14.14.3] |
| K15854 | *luxB*; alkanal monooxygenase beta chain [EC:1.14.14.3] |
| K06046 | *luxE*; long-chain-fatty-acid---luciferin-component ligase [EC:6.2.1.19] |
| K14579 | *nahAc, ndoB, nbzAc, dntAc*; naphthalene 1,2-dioxygenase subunit alpha [EC:1.14.12.12 1.14.12.23 1.14.12.24] |
| K14580 | *nahAd, ndoC, nbzAd, dntAd*; naphthalene 1,2-dioxygenase subunit beta [EC:1.14.12.12 1.14.12.23 1.14.12.24] |
| K14578 | *nahAb, nagAb, ndoA, nbzAb, dntAb*; naphthalene 1,2-dioxygenase ferredoxin component |
| K14581 | *nahAa, nagAa, ndoR, nbzAa, dntAa*; naphthalene 1,2-dioxygenase ferredoxin reductase component [EC:1.18.1.7] |
| K16249 | *dmpK, poxA, tomA0*; phenol/toluene 2-monooxygenase (NADH) P0/A0 |
| K16243 | *dmpL, poxB, tomA1*; phenol/toluene 2-monooxygenase (NADH) P1/A1 [EC:1.14.13.244 1.14.13.243] |
| K16244 | *dmpM, poxC, tomA2*; phenol/toluene 2-monooxygenase (NADH) P2/A2 [EC:1.14.13.244 1.14.13.243] |
| K16242 | *dmpN, poxD, tomA3*; phenol/toluene 2-monooxygenase (NADH) P3/A3 [EC:1.14.13.244 1.14.13.243] |
| K16245 | *dmpO, poxE, tomA4*; phenol/toluene 2-monooxygenase (NADH) P4/A4 [EC:1.14.13.244 1.14.13.243] |
| K16246 | *dmpP, poxF, tomA5*; phenol/toluene 2-monooxygenase (NADH) P5/A5 [EC:1.14.13.244 1.14.13.243] |
| K03268 | *todC1, bedC1, tcbAa*; benzene/toluene/chlorobenzene dioxygenase subunit alpha [EC:1.14.12.3 1.14.12.11 1.14.12.26] |
| K16268 | *todC2, bedC2, tcbAb*; benzene/toluene/chlorobenzene dioxygenase subunit beta [EC:1.14.12.3 1.14.12.11 1.14.12.26] |
| K18089 | *todB, tcbAc*; benzene/toluene/chlorobenzene dioxygenase ferredoxin component |
| K18090 | *todA, tcbAd*; benzene/toluene/chlorobenzene dioxygenase ferredoxin reductase component [EC:1.18.1.3 1.18.1.-] |
| K16269 | *todD, tcbB*; cis-1,2-dihydrobenzene-1,2-diol/chlorobenzene dihydrodiol dehydrogenase [EC:1.3.1.19 1.3.1.119] |
| K15760 | *tmoA, tbuA1, touA*; toluene monooxygenase system protein A [EC:1.14.13.236 1.14.13.-] |
| K15761 | *tmoB, tbuU, touB*; toluene monooxygenase system protein B [EC:1.14.13.236 1.14.13.-] |
| K15762 | *tmoC, tbuB, touC*; toluene monooxygenase system ferredoxin subunit |
| K15763 | *tmoD, tbuV, touD*; toluene monooxygenase system protein D [EC:1.14.13.236 1.14.13.-] |
| K15764 | *tmoE, tbuA2, touE*; toluene monooxygenase system protein E [EC:1.14.13.236 1.14.13.-] |
| K15765 | *tmoF, tbuC, touF*; toluene monooxygenase electron transfer component [EC:1.18.1.3] |
| K03380 | E1.14.13.7; phenol 2-monooxygenase (NADPH) [EC:1.14.13.7] |
| K15757 | *xylM*; toluene methyl-monooxygenase [EC:1.14.15.26] |
| K15758 | *xylA*; toluene methyl-monooxygenase electron transfer component [EC:1.18.1.3] |
| K00055 | E1.1.1.90; aryl-alcohol dehydrogenase [EC:1.1.1.90] |
| K00141 | *xylC*; benzaldehyde dehydrogenase (NAD) [EC:1.2.1.28] |

Table S3. Mean values of soil properties (WDPT, MED, and TPH) in 2014 and 1975 soil samples.

|  | MED (mol/L) | WDPT (s) | TPH  (mg/kg) |
| --- | --- | --- | --- |
| 14controlZero | 11.99 | 4000 | 15772 |
| 14control | 11.5 | 4000 | 15127 |
| 14W20_Rep1 | 6.17 | 2120 | 11357 |
| 14W20Nut_Rep1 | 5.27 | 1540 | 11160 |
| 14W20Surf_Rep1 | 4.91 | 500 | 10086 |
| 14W20NutSurf_Rep1 | 5.27 | 1280 | 10631 |
| 14W50_Rep1 | 8.86 | 4000 | 11338 |
| 75controlZero_Rep1 | 11.5 | 4000 | 2398 |
| 75control_Rep1 | 10.62 | 4000 | 3059 |
| 75W20_Rep1 | 3.66 | 1680 | 1931 |
| 75W20Nut_Rep1 | 3.48 | 160 | 578 |

Table S4. Top 20 abundant genera in 2014 contaminated soil samples.

| Genus | 14controlZero  _Rep1 | 14controlZero  _Rep2 | 14control  _Rep1 | 14control  _Rep2 | 14W20  _Rep1 | 14W20  _Rep2 | 14W50  _Rep1 | 14W50  _Rep2 | 14W20Nut  _Rep1 | 14W20Nut  _Rep2 | 14W20Surf  _Rep1 | 14W20Surf  _Rep2 | 14W20NutSurf  _Rep1 | 14W20NutSurf  _Rep2 |
| --- | --- | --- | --- | --- | --- | --- | --- | --- | --- | --- | --- | --- | --- | --- |
| JG3-KF-CM45_ge | 1.03 | 1.09 | 1.79 | 1.59 | 8.13 | 6.90 | 8.78 | 9.97 | 20.47 | 14.78 | 11.38 | 11.53 | 13.50 | 15.29 |
| KCM-B-2 | 0.01 | 0.48 | 0.01 | 0.02 | 3.64 | 2.89 | 20.76 | 18.47 | 11.85 | 25.20 | 9.55 | 3.77 | 8.54 | 5.35 |
| Immundisolibacter | 0.03 | 0.03 | 0.01 | 0.03 | 7.01 | 8.29 | 2.91 | 2.10 | 13.88 | 17.90 | 20.52 | 7.02 | 15.24 | 14.03 |
| Microbacteriaceae_unclassified | 0.52 | 0.56 | 0.31 | 0.51 | 17.51 | 17.81 | 1.82 | 1.63 | 6.83 | 4.15 | 11.64 | 22.58 | 6.74 | 8.66 |
| Actinobacteria_unclassified | 0.33 | 0.28 | 0.75 | 0.57 | 0.57 | 0.40 | 1.94 | 1.66 | 12.62 | 11.32 | 1.57 | 1.81 | 9.94 | 9.96 |
| Alkanindiges | 8.64 | 9.49 | 9.27 | 10.90 | 0.01 | 0.01 | 0.00 | 0.00 | 0.00 | 0.01 | 0.00 | 0.00 | 0.01 | 0.00 |
| Pseudomonas | 1.83 | 2.32 | 2.15 | 2.36 | 0.12 | 0.14 | 11.52 | 13.77 | 0.71 | 0.31 | 0.14 | 0.04 | 0.24 | 0.24 |
| Luteimonas | 0.01 | 0.00 | 0.01 | 0.01 | 6.77 | 6.61 | 0.01 | 0.01 | 1.74 | 2.12 | 3.03 | 3.08 | 5.12 | 3.08 |
| uncultured | 4.07 | 3.90 | 3.06 | 3.06 | 2.40 | 2.73 | 2.83 | 2.70 | 0.32 | 0.43 | 1.90 | 1.35 | 1.61 | 0.93 |
| Saccharimonadaceae_ge | 0.11 | 0.15 | 0.05 | 0.04 | 10.97 | 10.48 | 1.93 | 1.78 | 0.44 | 0.39 | 1.02 | 0.72 | 0.78 | 0.70 |
| Mycobacterium | 0.33 | 0.73 | 0.22 | 0.23 | 0.62 | 0.83 | 0.00 | 0.03 | 1.03 | 0.76 | 4.72 | 3.85 | 8.37 | 7.83 |
| Solimonadaceae_unclassified7 | 0.03 | 0.01 | 0.01 | 0.01 | 6.24 | 7.27 | 0.04 | 0.03 | 0.51 | 0.58 | 3.65 | 8.47 | 1.11 | 0.77 |
| Solimonadaceae_unclassified | 0.00 | 0.01 | 0.02 | 0.00 | 4.19 | 4.08 | 2.45 | 1.44 | 0.89 | 1.23 | 3.92 | 5.88 | 1.71 | 0.97 |
| Opitutaceae_unclassified99 | 0.01 | 0.01 | 0.01 | 0.01 | 5.01 | 5.96 | 0.35 | 0.33 | 0.88 | 1.81 | 3.24 | 2.50 | 1.56 | 1.06 |
| Rhodobacteraceae_unclassified88 | 0.00 | 0.01 | 0.00 | 0.00 | 0.16 | 0.17 | 7.59 | 7.76 | 0.41 | 0.19 | 0.24 | 0.06 | 0.80 | 0.86 |
| Nocardioides | 1.01 | 0.84 | 1.15 | 1.20 | 1.09 | 1.00 | 0.25 | 0.18 | 2.54 | 1.08 | 1.03 | 1.88 | 0.97 | 2.69 |
| Altererythrobacter | 0.19 | 0.27 | 0.41 | 0.36 | 1.45 | 1.55 | 1.75 | 2.01 | 1.04 | 1.46 | 1.10 | 1.01 | 1.98 | 1.45 |
| uncultured_ge | 2.91 | 2.54 | 2.50 | 2.76 | 1.09 | 0.91 | 0.17 | 0.15 | 0.40 | 0.25 | 0.26 | 0.18 | 0.86 | 0.71 |
| Streptomyces69 | 4.24 | 4.39 | 3.03 | 2.83 | 0.19 | 0.14 | 0.04 | 0.02 | 0.04 | 0.02 | 0.10 | 0.20 | 0.02 | 0.06 |
| Parachlamydiaceae_unclassified | 0.00 | 0.00 | 0.01 | 0.02 | 1.75 | 1.50 | 0.00 | 0.01 | 1.11 | 0.58 | 1.46 | 3.54 | 1.00 | 1.78 |

Table S5. Top 20 abundant genera in 1975 contaminated soil samples.

| Genus | 75controlZero_  Rep1 | 75controlZero_  Rep2 | 75control_  Rep1 | 75control_  Rep2 | 75W20_  Rep1 | 75W20_  Rep2 | 75W20Nut_  Rep1 | 75W20Nut_  Rep2 |
| --- | --- | --- | --- | --- | --- | --- | --- | --- |
| uncultured_ge | 9.76 | 6.61 | 8.66 | 9.27 | 6.80 | 7.02 | 7.94 | 8.80 |
| JG3-KF-CM45_ge | 2.00 | 2.52 | 2.57 | 2.40 | 4.93 | 5.07 | 5.34 | 5.22 |
| Saccharimonadales_ge | 4.37 | 3.10 | 2.47 | 2.51 | 0.68 | 0.97 | 4.62 | 4.89 |
| uncultured | 0.65 | 0.27 | 0.38 | 0.46 | 7.37 | 7.38 | 3.03 | 2.56 |
| Kocuria63 | 5.14 | 4.47 | 5.59 | 6.27 | 0.07 | 0.09 | 0.01 | 0.03 |
| 67-4_ge | 0.01 | 0.02 | 0.00 | 0.00 | 4.99 | 4.16 | 5.89 | 6.52 |
| Sphingomonas92 | 0.51 | 0.67 | 0.62 | 0.67 | 2.05 | 1.98 | 7.11 | 6.87 |
| Gitt-GS-36_ge | 0.16 | 0.12 | 0.08 | 0.05 | 4.42 | 5.04 | 5.01 | 5.54 |
| Longimicrobiaceae_ge | 3.30 | 5.53 | 5.16 | 5.85 | 0.12 | 0.13 | 0.17 | 0.13 |
| Microbacteriaceae_unclassified | 1.02 | 0.90 | 0.88 | 1.19 | 4.98 | 4.75 | 2.16 | 2.26 |
| Pontibacter | 3.25 | 4.27 | 4.15 | 3.86 | 0.00 | 0.01 | 0.00 | 0.00 |
| Truepera | 0.21 | 0.17 | 0.19 | 0.19 | 1.33 | 0.95 | 5.15 | 6.06 |
| Saccharimonadaceae_ge | 0.54 | 0.48 | 0.32 | 0.43 | 0.96 | 0.79 | 5.28 | 4.43 |
| A4b_ge | 0.02 | 0.01 | 0.00 | 0.00 | 3.84 | 3.85 | 2.47 | 2.81 |
| Blastococcus9 | 2.47 | 2.96 | 3.46 | 2.86 | 0.09 | 0.07 | 0.08 | 0.10 |
| JG3-KF-CM66_ge | 0.05 | 0.01 | 0.01 | 0.02 | 0.77 | 0.93 | 5.29 | 5.01 |
| Blastococcus8 | 2.45 | 3.11 | 3.08 | 2.74 | 0.11 | 0.12 | 0.23 | 0.14 |
| Marmoricola95 | 3.14 | 2.74 | 2.33 | 2.34 | 0.01 | 0.02 | 0.01 | 0.05 |
| Iamia | 0.00 | 0.00 | 0.00 | 0.00 | 4.53 | 4.47 | 0.50 | 0.55 |
| Dietzia | 2.29 | 2.13 | 2.38 | 2.45 | 0.14 | 0.12 | 0.21 | 0.20 |
