# Supplementary figures and images for: Biostimulation Shaped Microbial Communities in Oil-contaminated Desert Soils
Source: Curr Microbiol. 2026 Feb 10;83(4):167. doi: 10.1007/s00284-026-04756-x (PMC12891259; doi:10.1007/s00284-026-04756-x)

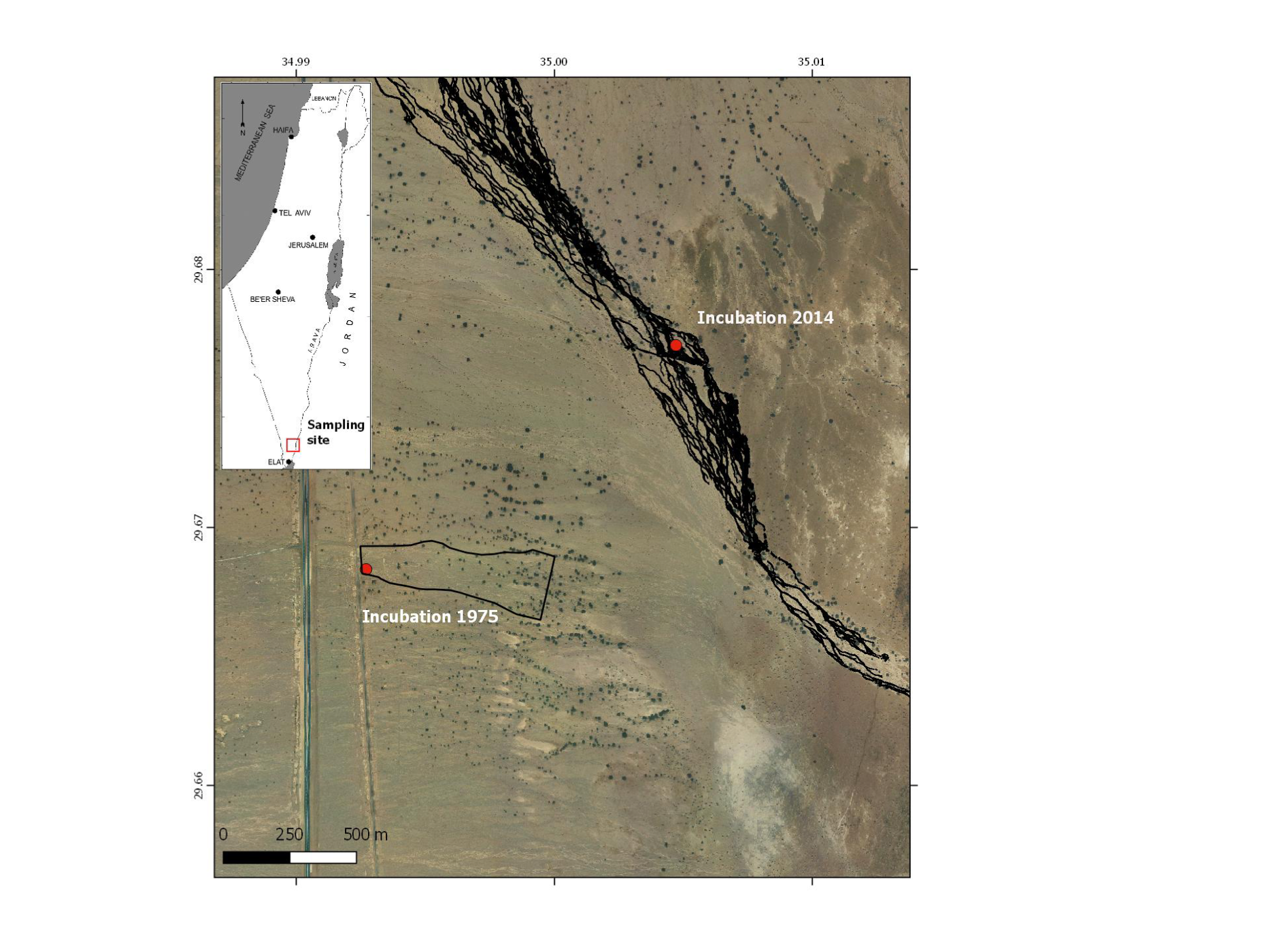

Supplement: Supplementary file 2 — Supplementary Material 2 [file 284_2026_4756_MOESM2_ESM.tiff]

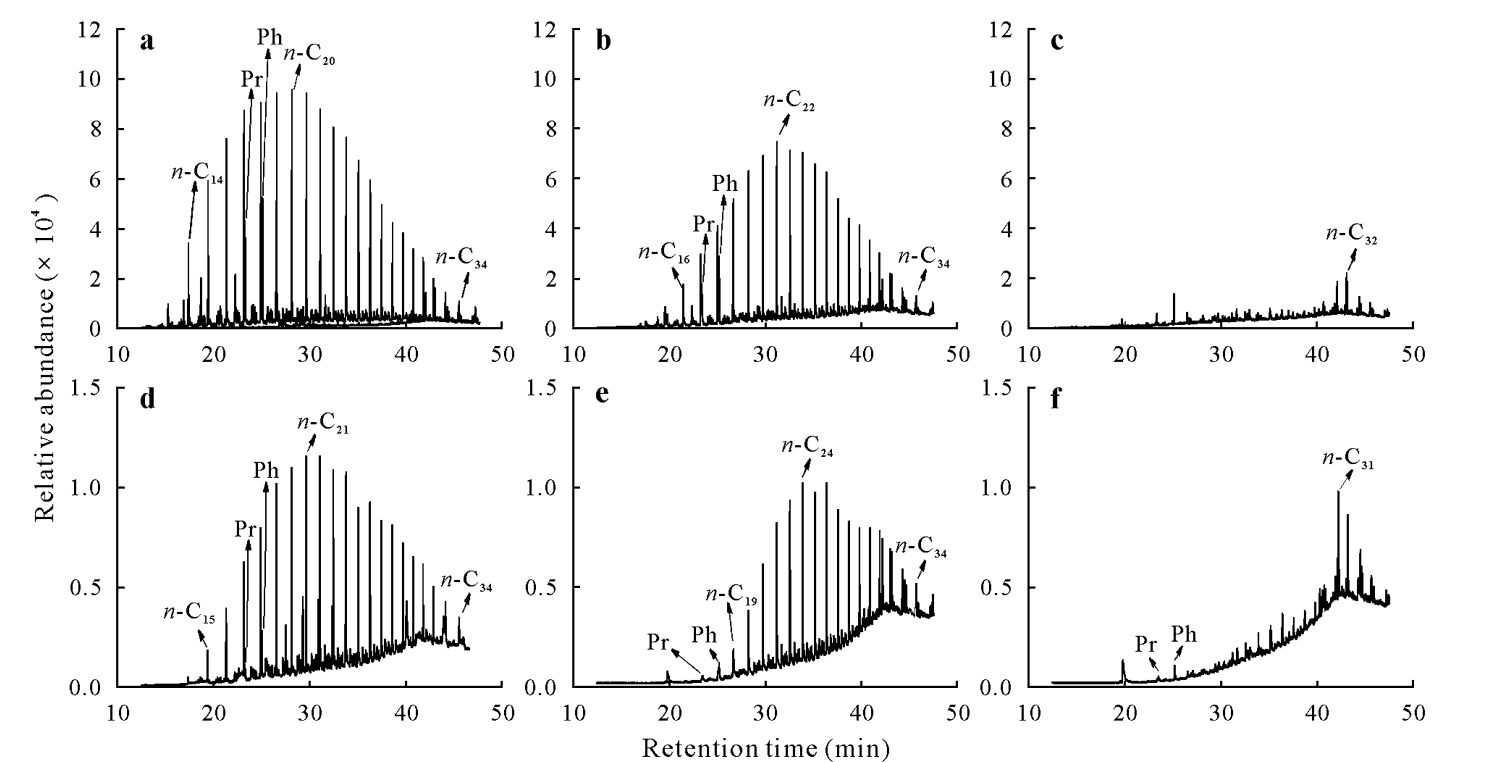

Supplement: Supplementary file 3 — Supplementary Material 3 [file 284_2026_4756_MOESM3_ESM.tiff]

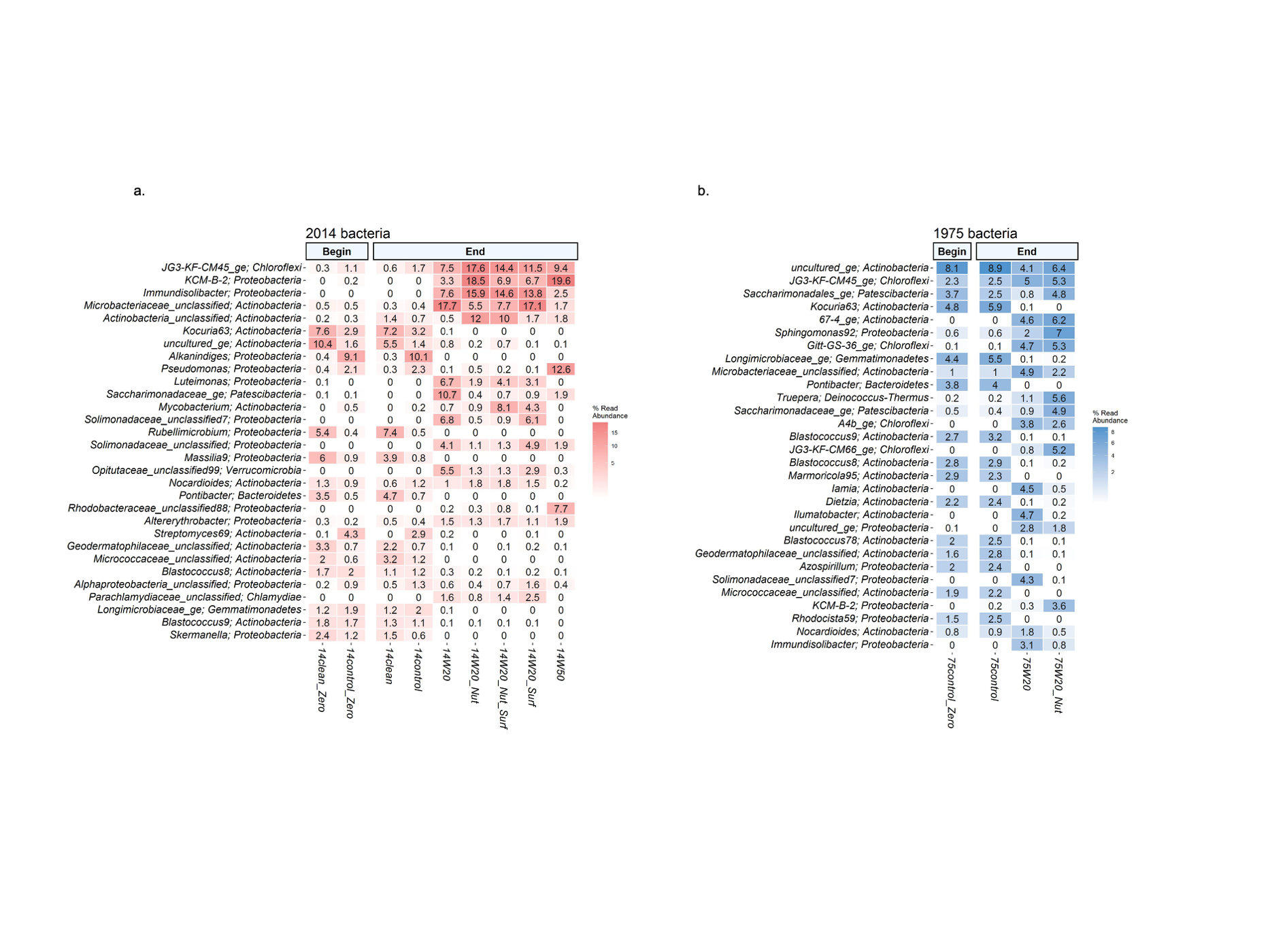

Supplement: Supplementary file 4 — Supplementary Material 4 [file 284_2026_4756_MOESM4_ESM.tiff]

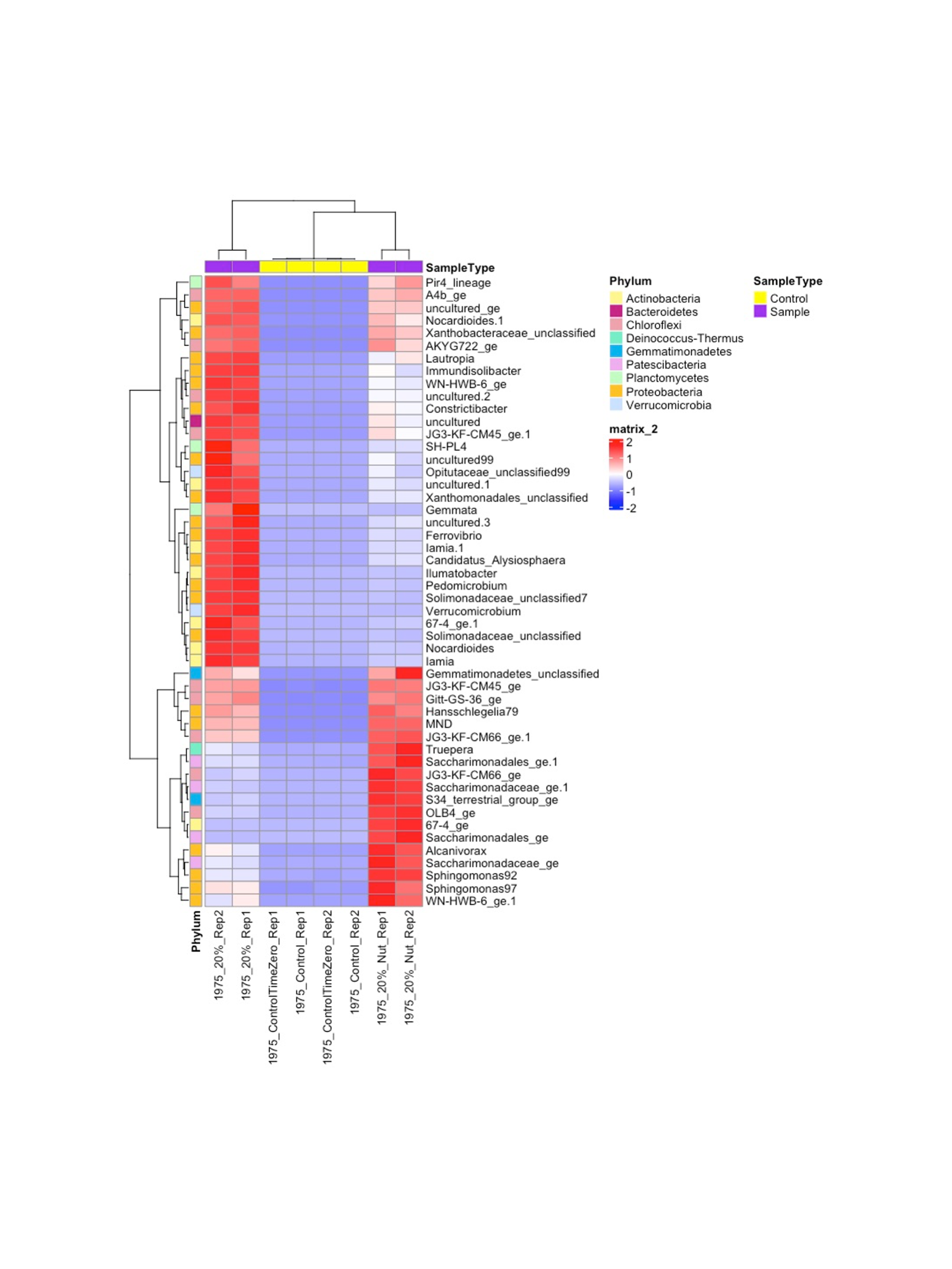

Supplement: Supplementary file 5 — Supplementary Material 5 [file 284_2026_4756_MOESM5_ESM.tiff]

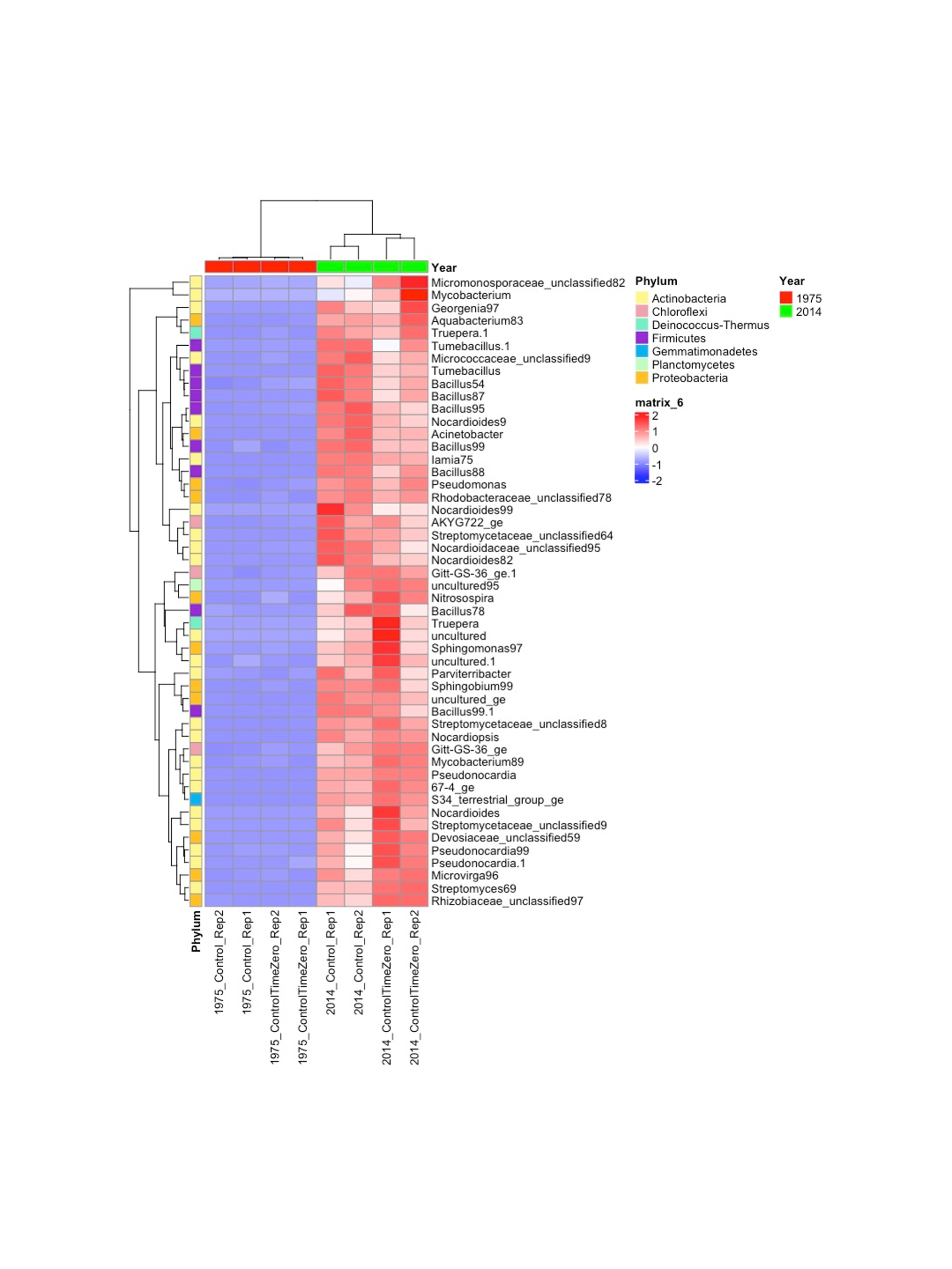

Supplement: Supplementary file 6 — Supplementary Material 6 [file 284_2026_4756_MOESM6_ESM.tiff]

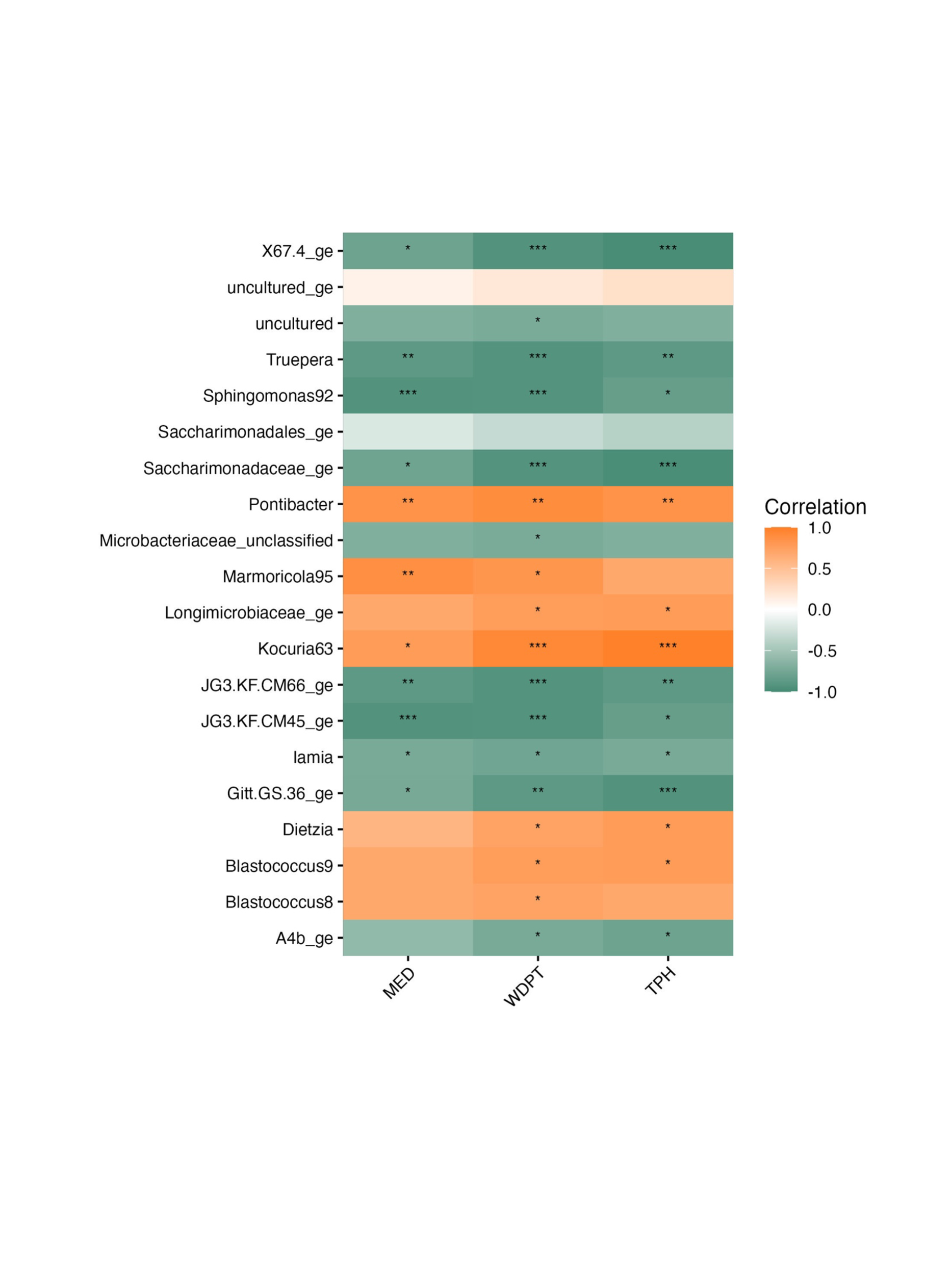

Supplement: Supplementary file 7 — Supplementary Material 7 [file 284_2026_4756_MOESM7_ESM.tiff]

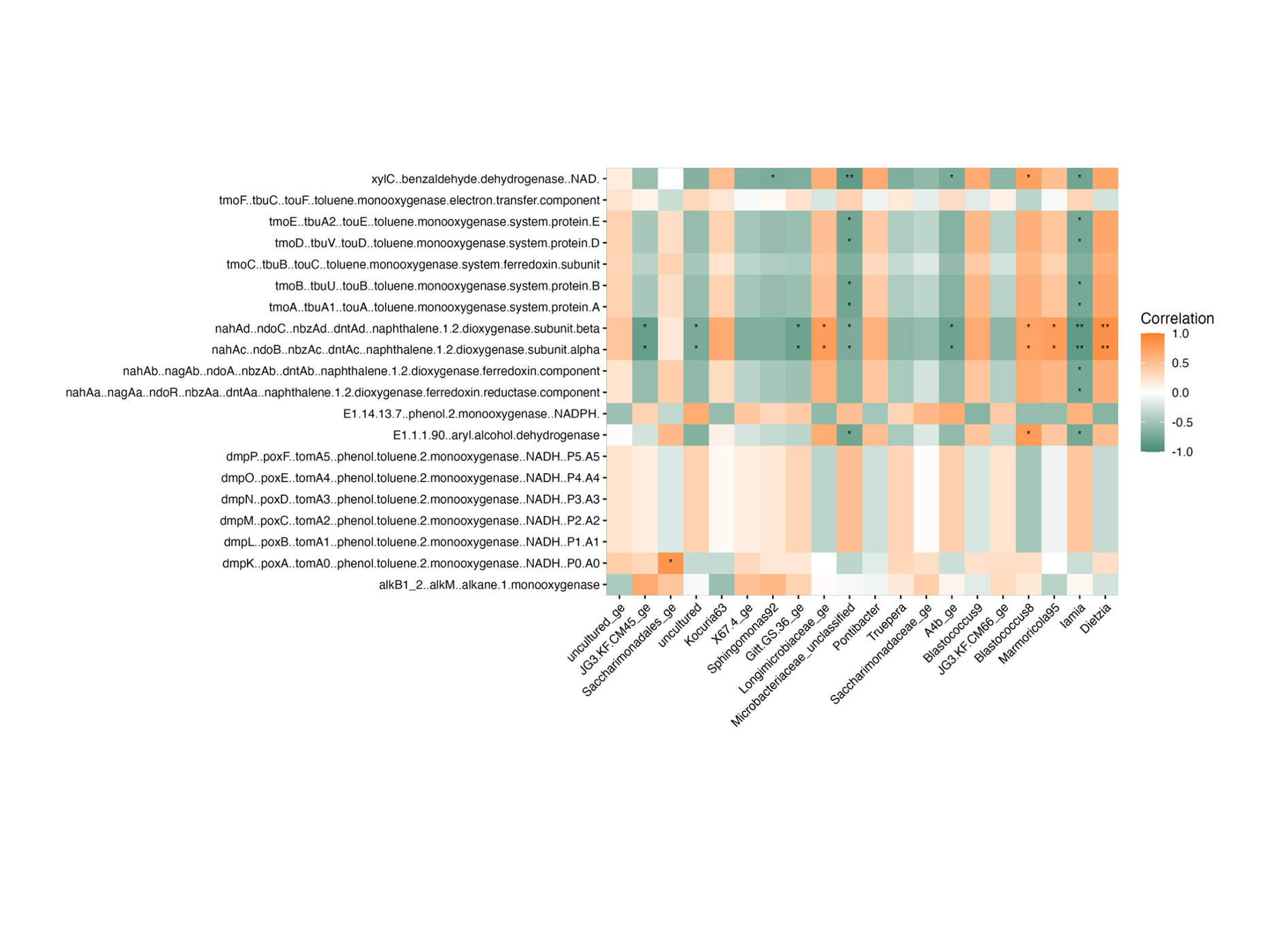

Supplement: Supplementary file 8 — Supplementary Material 8 [file 284_2026_4756_MOESM8_ESM.tiff]

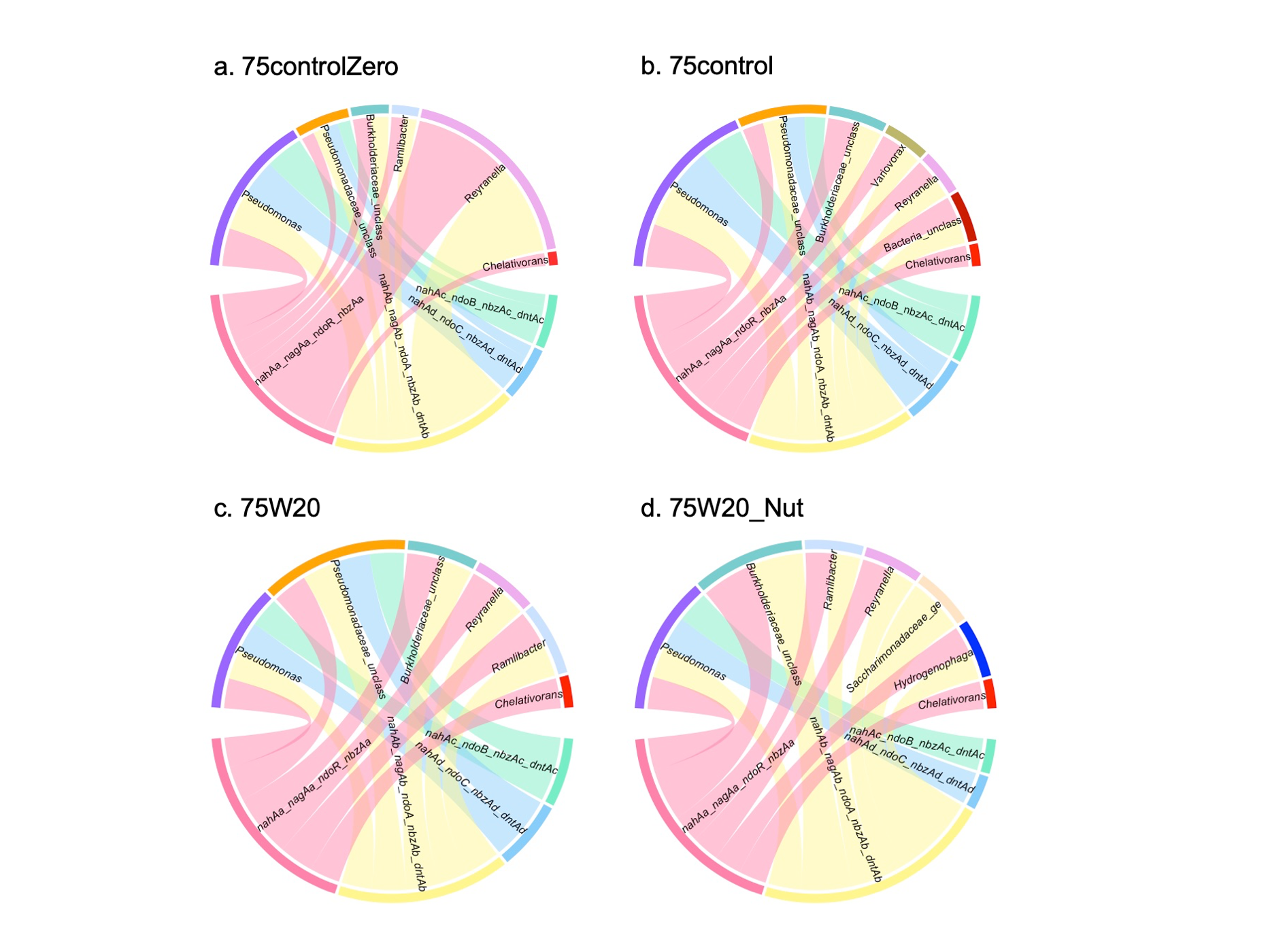

Supplement: Supplementary file 9 — Supplementary Material 9 [file 284_2026_4756_MOESM9_ESM.tiff]

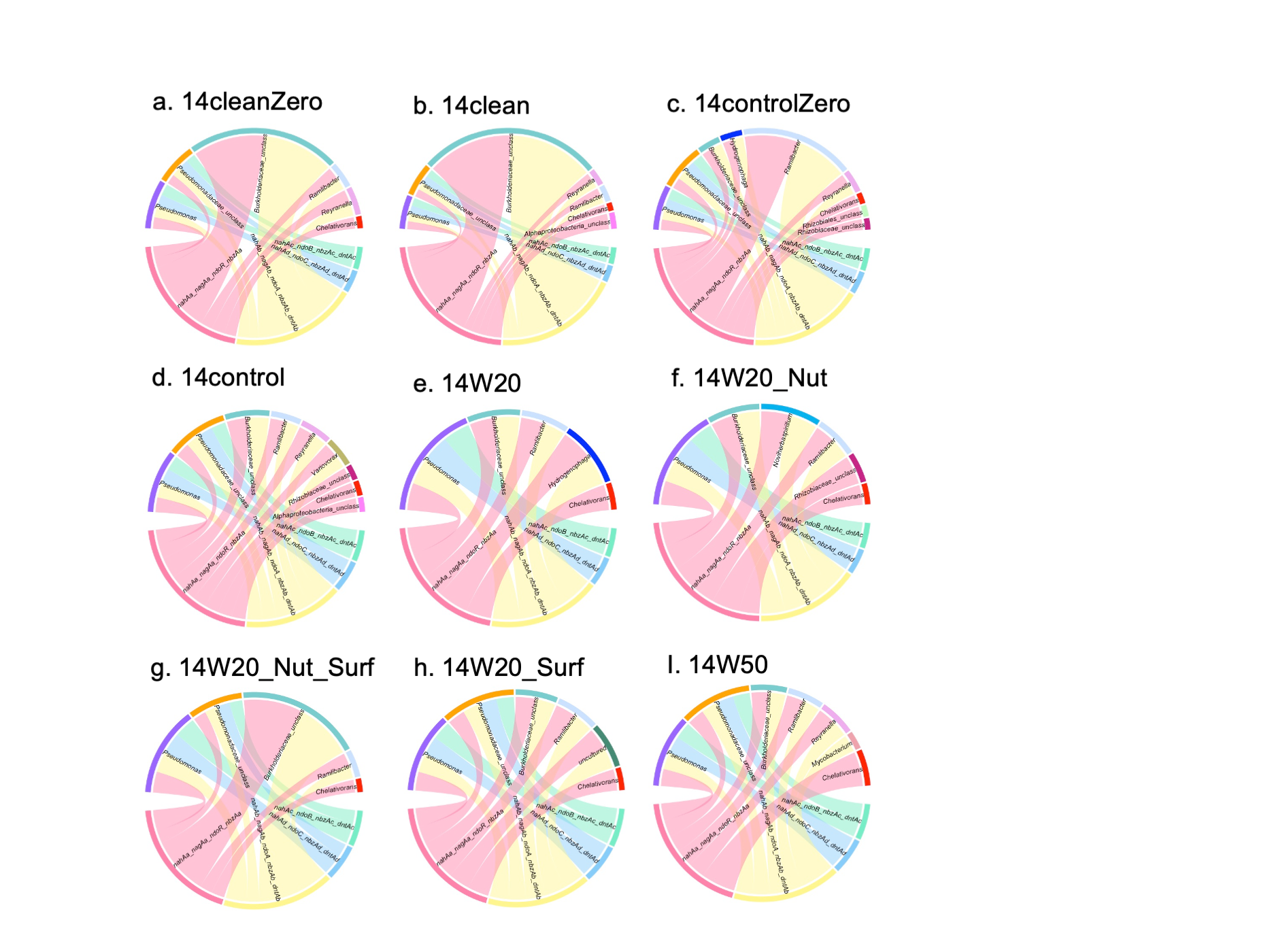

Supplement: Supplementary file 10 — Supplementary Material 10 [file 284_2026_4756_MOESM10_ESM.tiff]

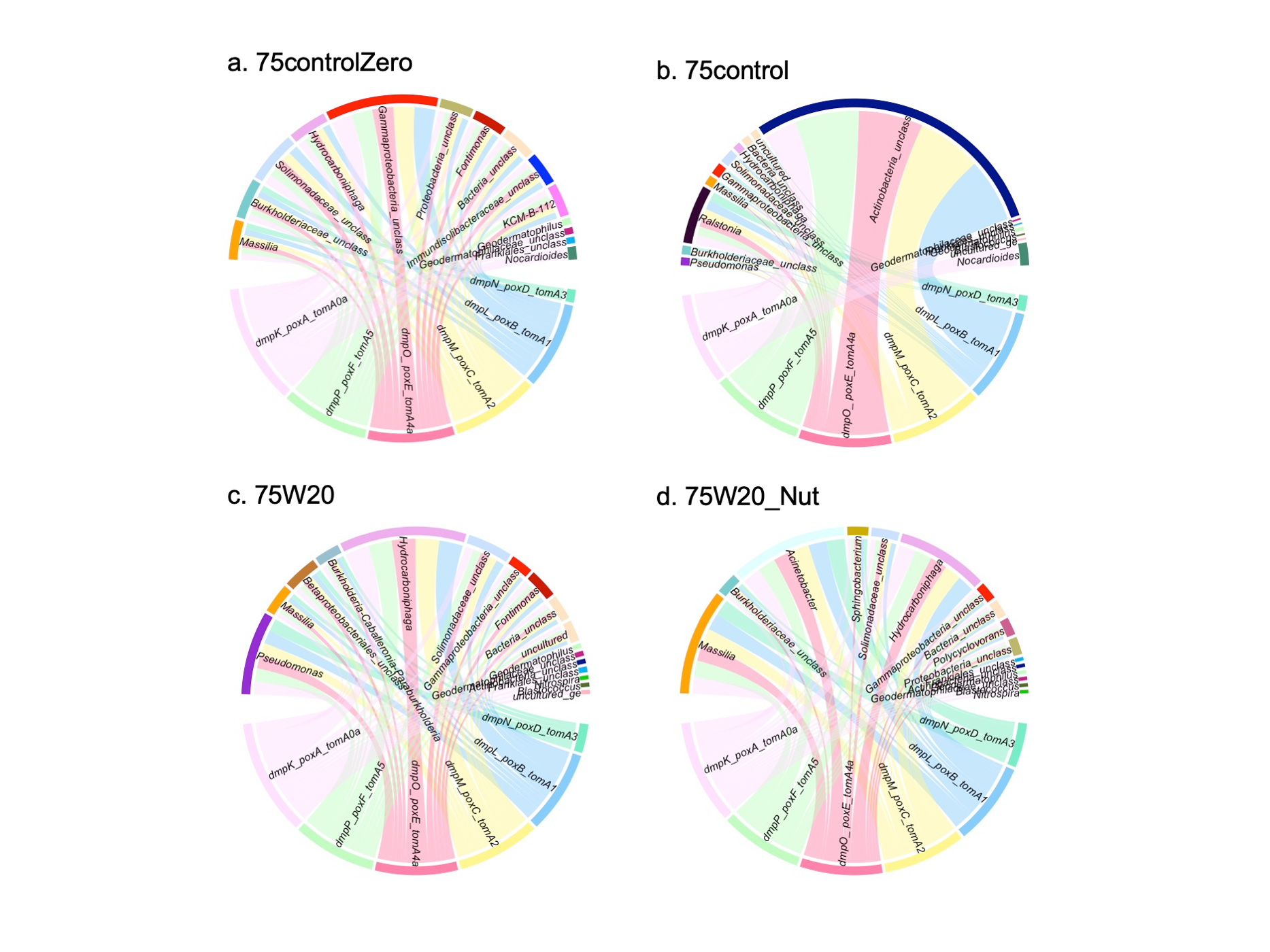

Supplement: Supplementary file 11 — Supplementary Material 11 [file 284_2026_4756_MOESM11_ESM.tiff]

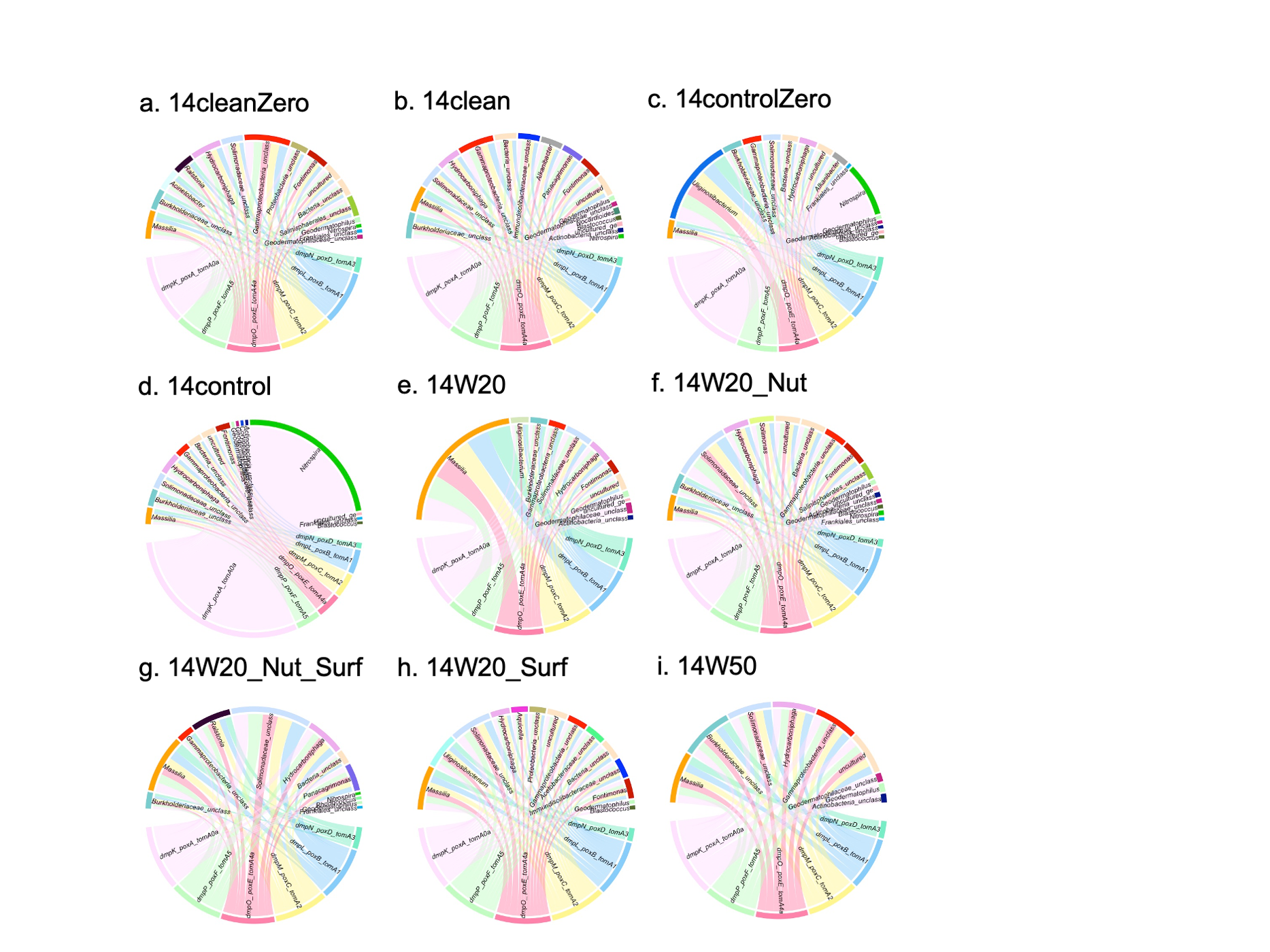

Supplement: Supplementary file 12 — Supplementary Material 12 [file 284_2026_4756_MOESM12_ESM.tiff]
