# Supplementary material for: Biostimulation Shaped Microbial Communities in Oil-contaminated Desert Soils
Source: Curr Microbiol. 2026 Feb 10;83(4):167. doi: 10.1007/s00284-026-04756-x (PMC12891259; doi:10.1007/s00284-026-04756-x)
Supplement: Supplementary file 13 — Supplementary Material 13 [file 284_2026_4756_MOESM13_ESM.docx]

Supporting Information for

**Biostimulation Shaped Microbial Communities in Oil-contaminated Desert Soils**

Zheng Li^1,2*, a^, Mitiku Mihiret Seyoum^3^, Ravid Rosenzweig^2^, Faina Gelman^2^, Zeev Ronen^1*^

1 Zuckerberg Institute for Water Research, Jacob Blaustein Institutes for Desert Research, Ben-Gurion University of the Negev, 84990 Sede Boqer Campus, Israel

2 Geological Survey of Israel, 32 Yeshayahu Leibowitz St., 9692100 Jerusalem, Israel

3 Department of Crop, Soil, and Environmental Sciences, University of Arkansas, Fayetteville, AR, USA

a- present address: Department of Environmental Sciences, University of California, Riverside, CA, USA

* Correspondence:

[zli@ucr.edu](mailto:zli@ucr.edu)

[zeevrone@bgu.ac.il](mailto:zeevrone@bgu.ac.il)


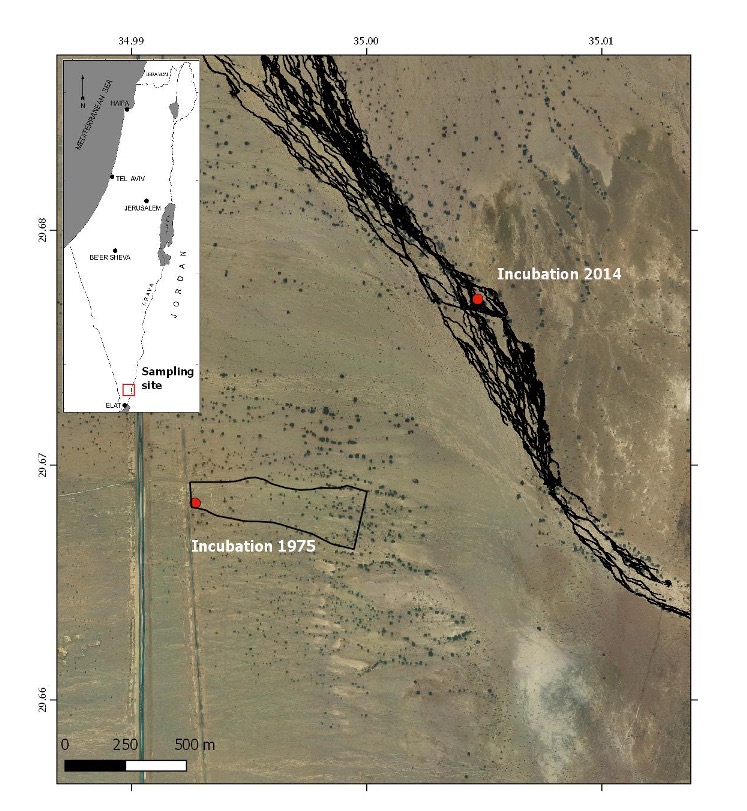


**Fig. S1** Map showing the two soil sampling points (triangles) at the 1975 and 2014 oil spill sites in the hyper-arid Evrona Nature Reserve, southern Israel. The black lines indicate the streams contaminated during the 2014 oil spill event and the area contaminated during the 1975 oil spill event.


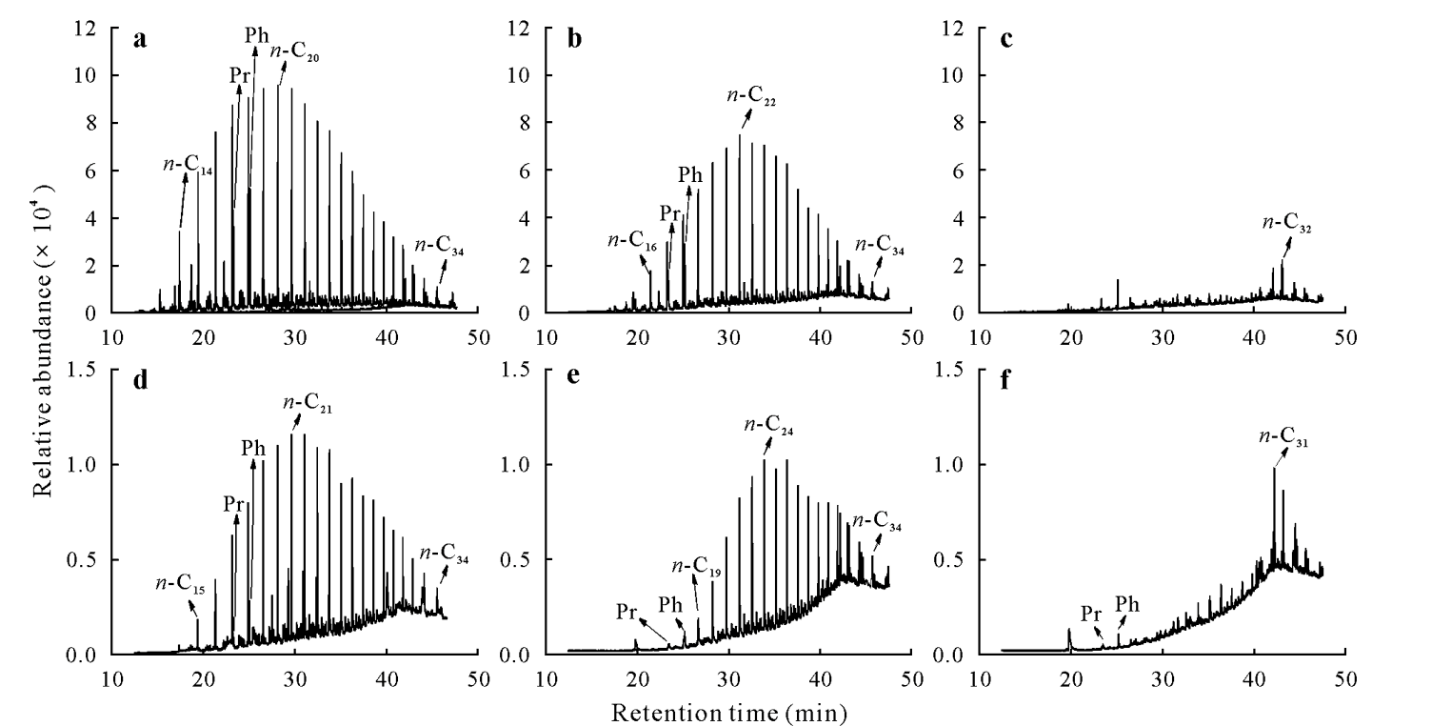


**Fig. S2** Gas chromatography-mass spectrometry (GC-MS) chromatograms of hydrocarbons extracted from the soils collected from the 2014 (a–c) and 1975 (d–f) oil spill sites in the hyper-arid Evrona Nature Reserve, southern Israel. Untreated (control) soils before (a and d) and after (b and e) one year of incubation and soils treated with water (20% water saturation) and nutrients for one year (c and f) are presented. Pr = pristane; Ph = phytane. Reproduce with permission from Elsevier B.V. and Science Press; Order number: 6143150662802”.


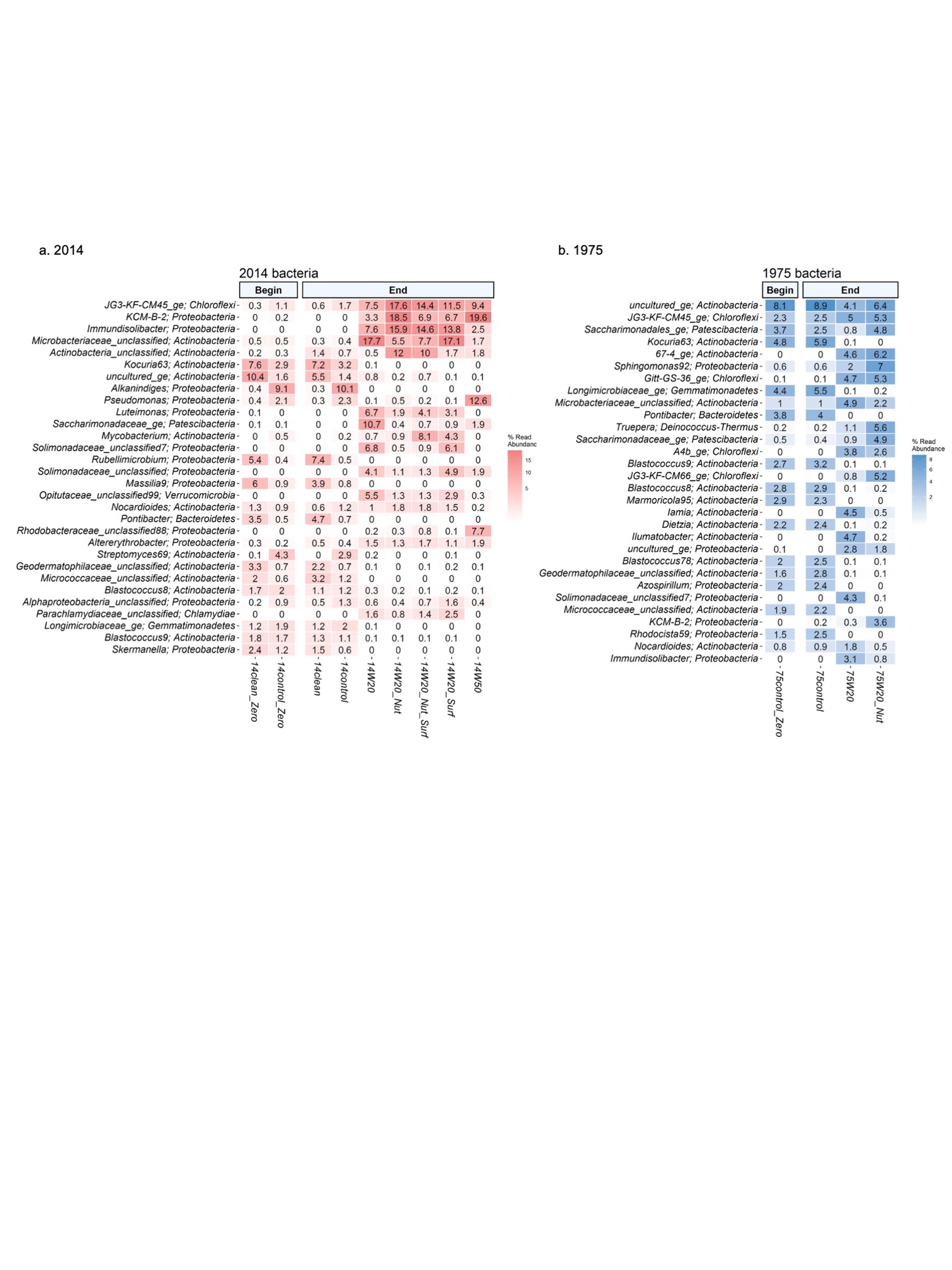


**Fig. S3** Heatmap of the abundance of top 30 abundant OTUs of the bacterial populations in 2014 (a) and 1975 (b) soil samples at the beginning and the end of the incubation.


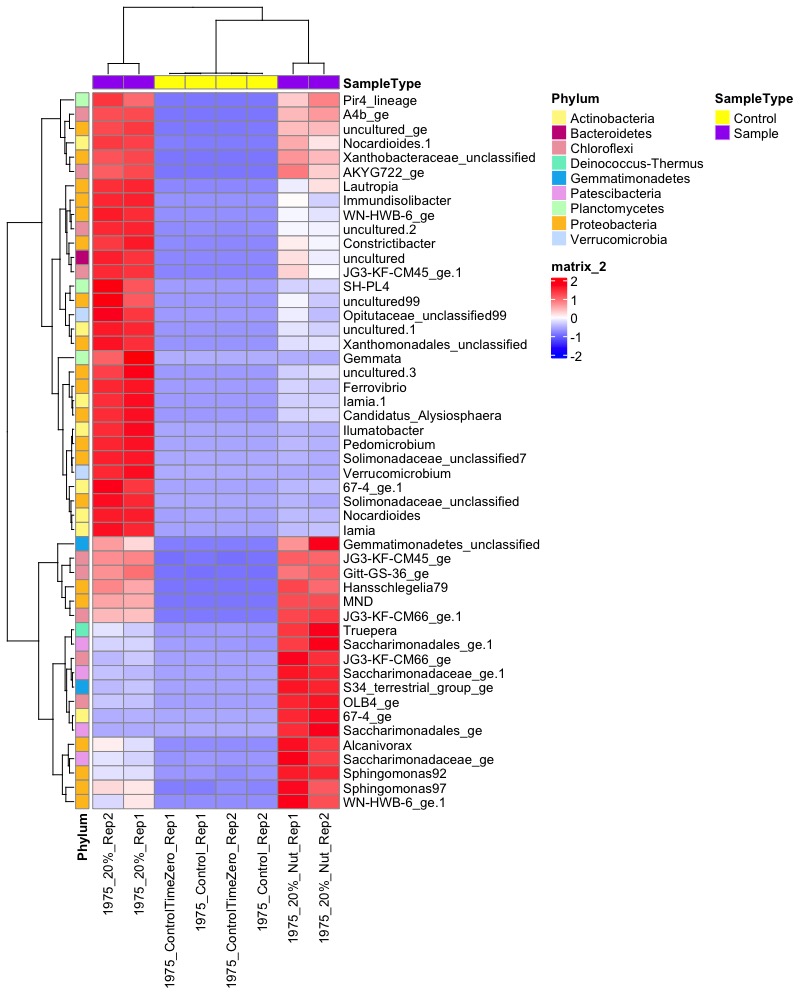


**Fig. S4** The top 50 differentially abundant taxa between the 1975 control soils and other 1975 soil treatments (as determined by DESeq analysis).


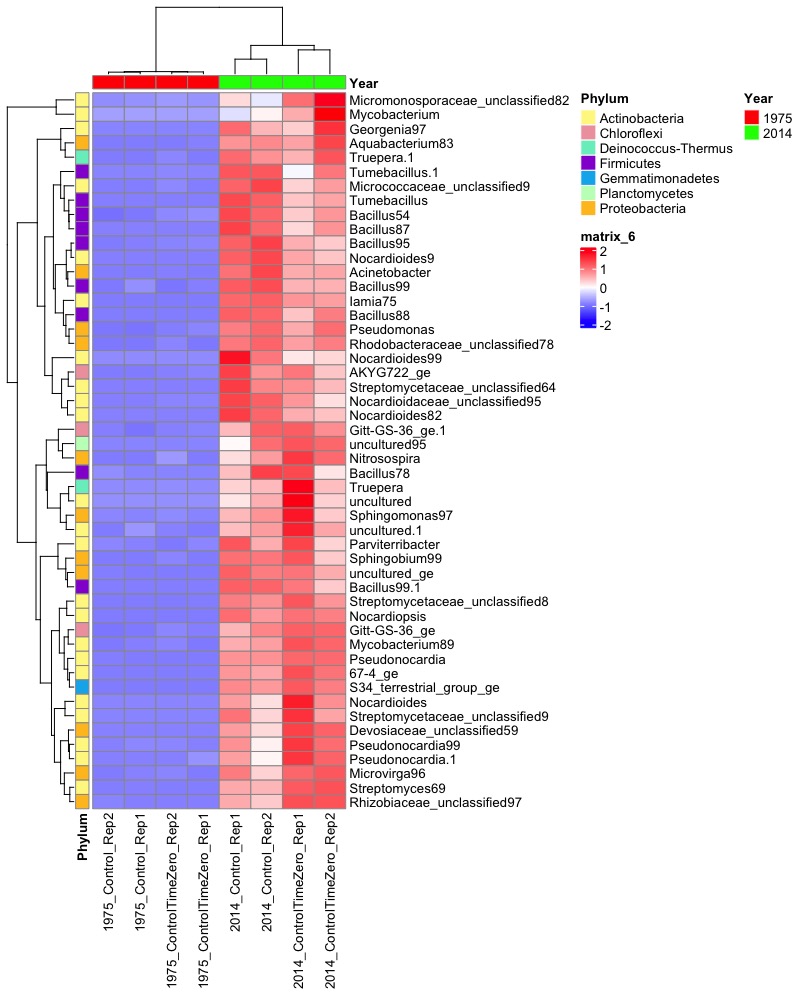


**Fig. S5** The top 50 differentially abundant taxa between the 1975 control and 2014 control soils (as determined by DESeq analysis).


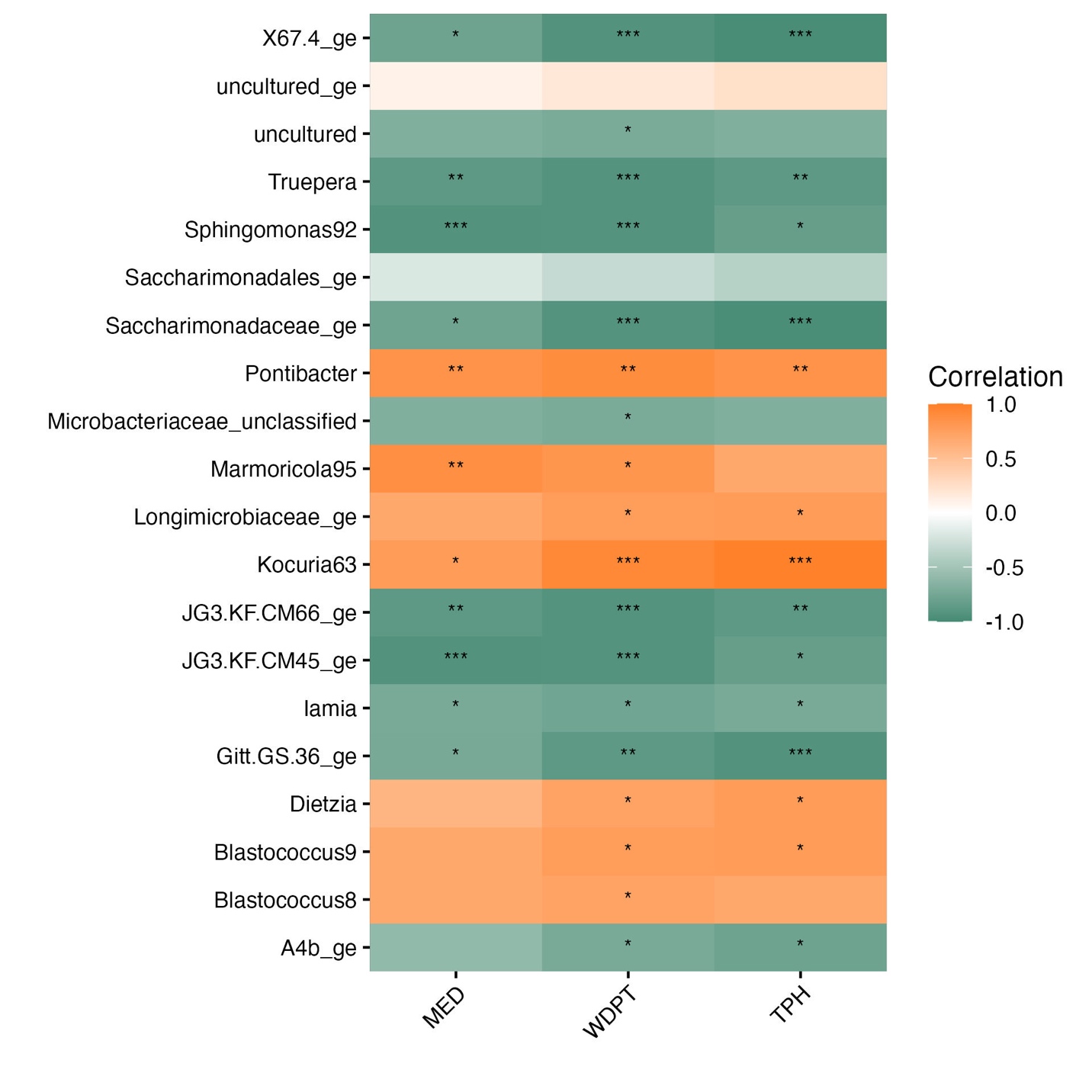


**Fig. S6** Heatmap of the correlations between the abundance of top 20 genera and soil properties (WDPT, MED, and TPH) in the 1975 contaminated. “*” indicates p < 0.05, “**” indicates p < 0.01 and “***” indicates p < 0.001. Statistical analyses are exploratory due to the absence of biological replication


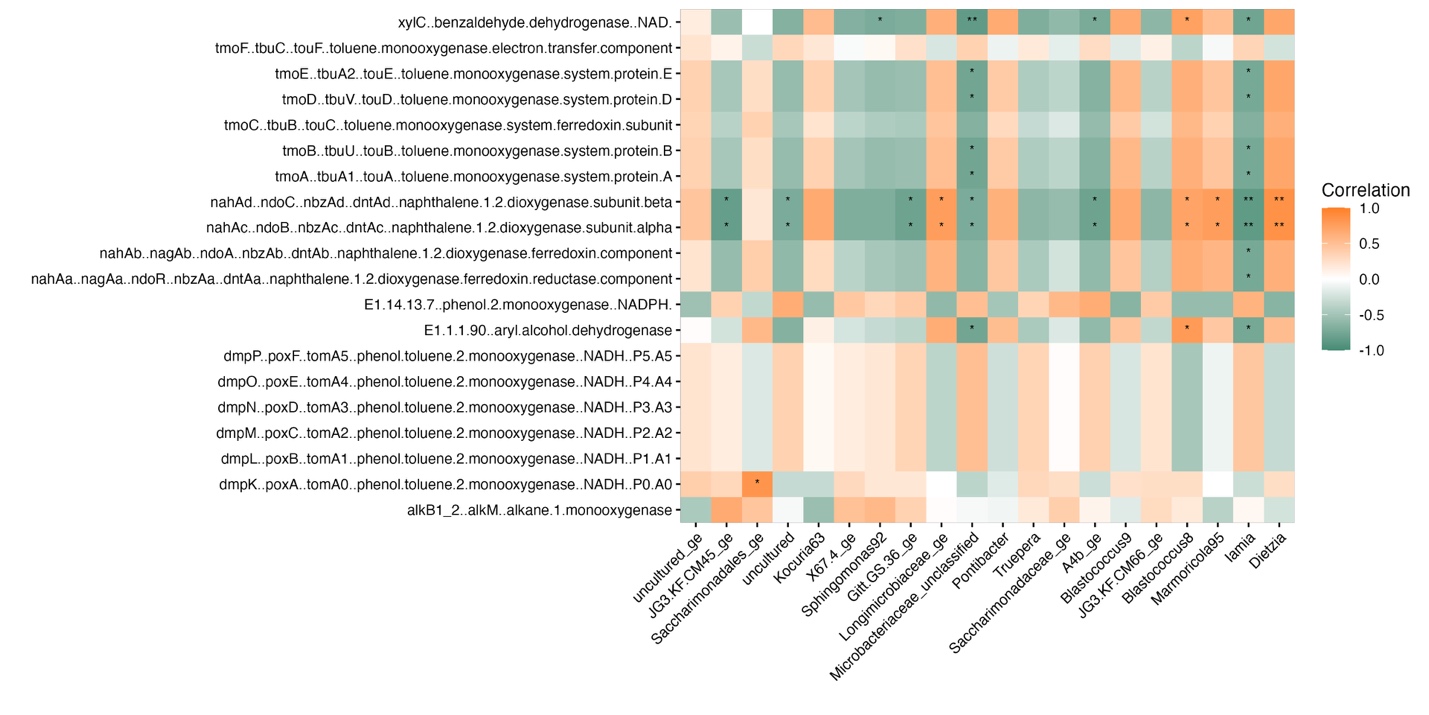


**Fig. S7** Heatmap of the correlations between the abundance of top 20 genera and functions predicted by PICRUSt2 in the 1975 contaminated soils. “*” indicates p < 0.05, “**” indicates p < 0.01 and “***” indicates p < 0.001. Statistical analyses are exploratory due to the absence of biological replication


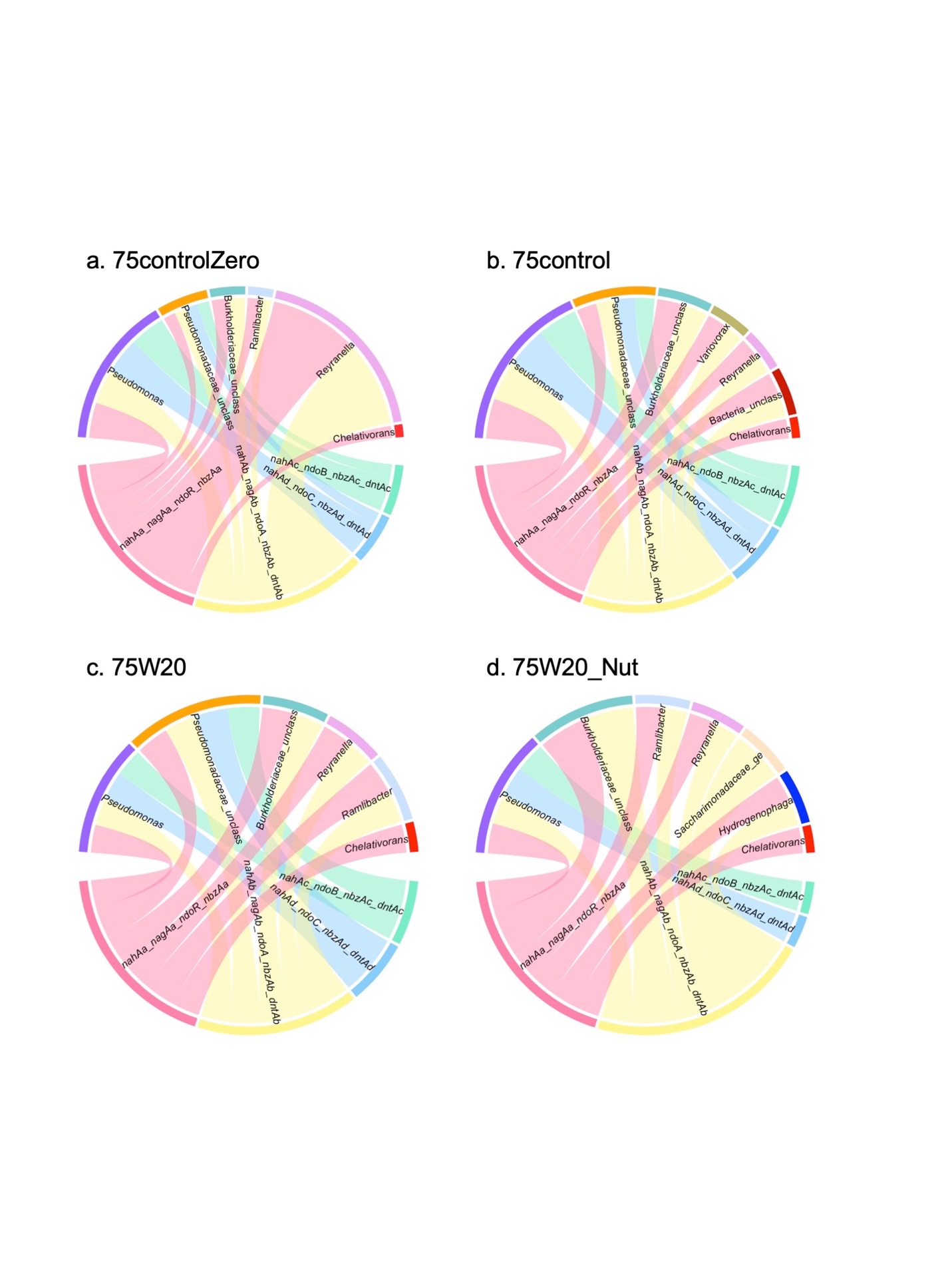


**Fig. S8** Phylotypes associated with naphthalene dioxygenase genes in the 75controlZero (a), 75control (b), 75W20 (c), 75W20_Nut (d). To limit the number of phylotypes on each figure, the relative abundance threshold was set to > 0.005.


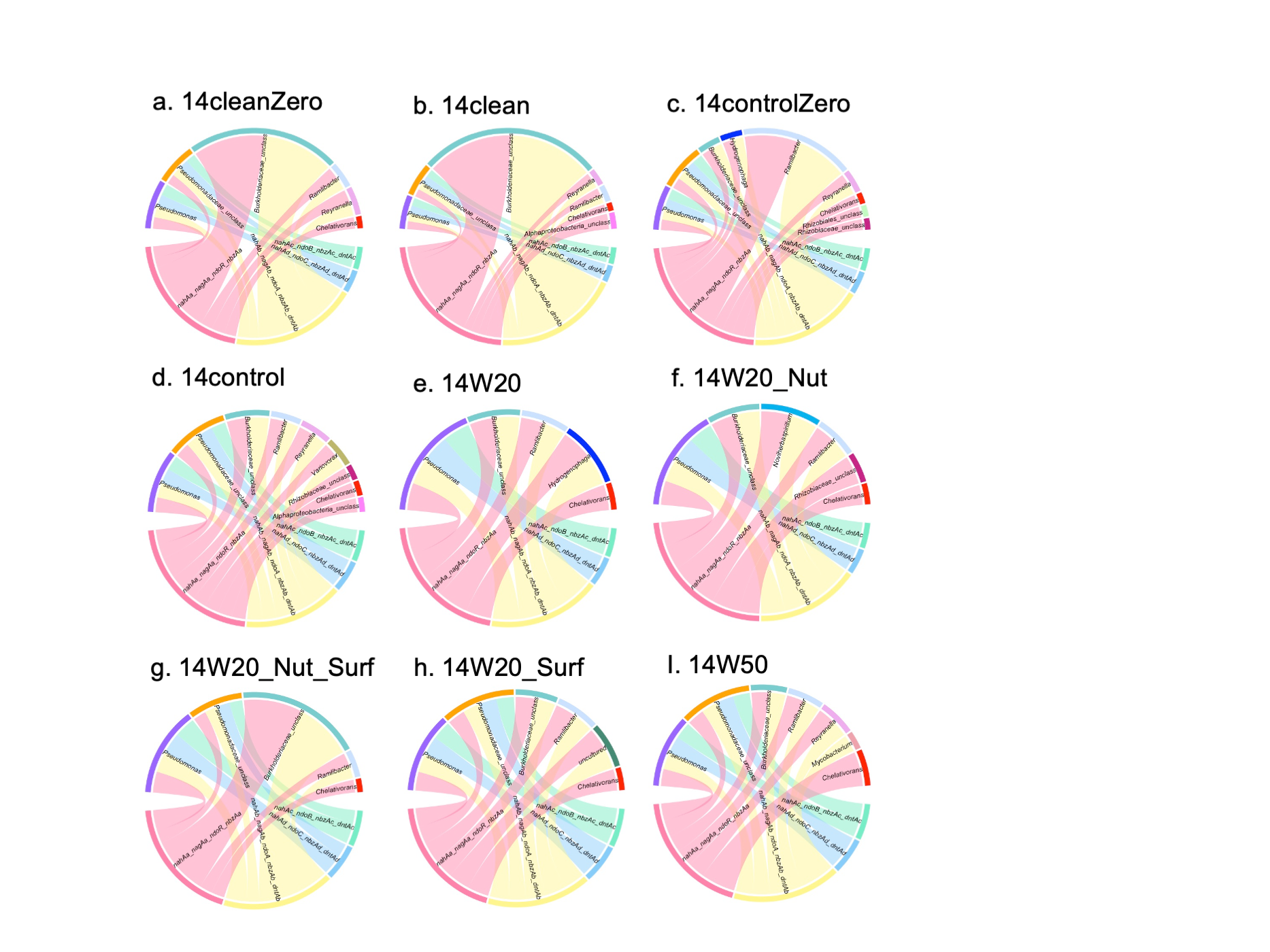


**Fig. S9** Circus plots illustrating the associations between phylotypes and naphthalene dioxygenase genes (*nahAc*) across treatments in the 2014 soils: 14cleanZero (a), 14clean (b), 14controlZero (c), 14control (d), 14W20 (e), 14W20Nut (f), 14W20NutSurf (g), 14W20Surf (h), 14W50 (i). To limit the number of phylotypes on each figure, the relative abundance threshold was set to > 0.005.


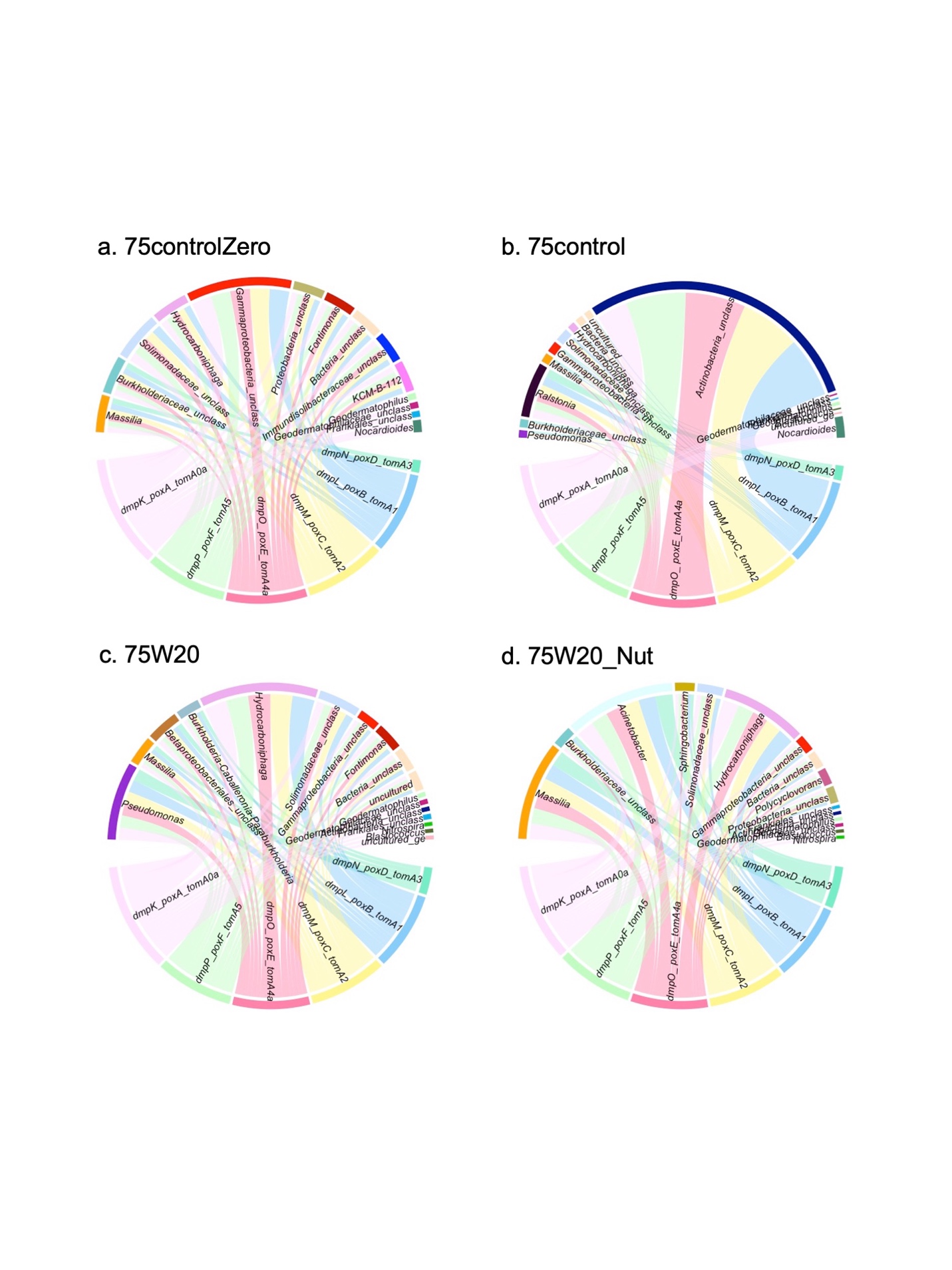


**Fig. S10** Phylotypes associated with phenol/toluene monooxygenase genes in the 75controlZero (a), 75control (b), 75W20 (c), 75W20_Nut (d). To limit the number of phylotypes on each figure, the relative abundance threshold was set to > 0.005


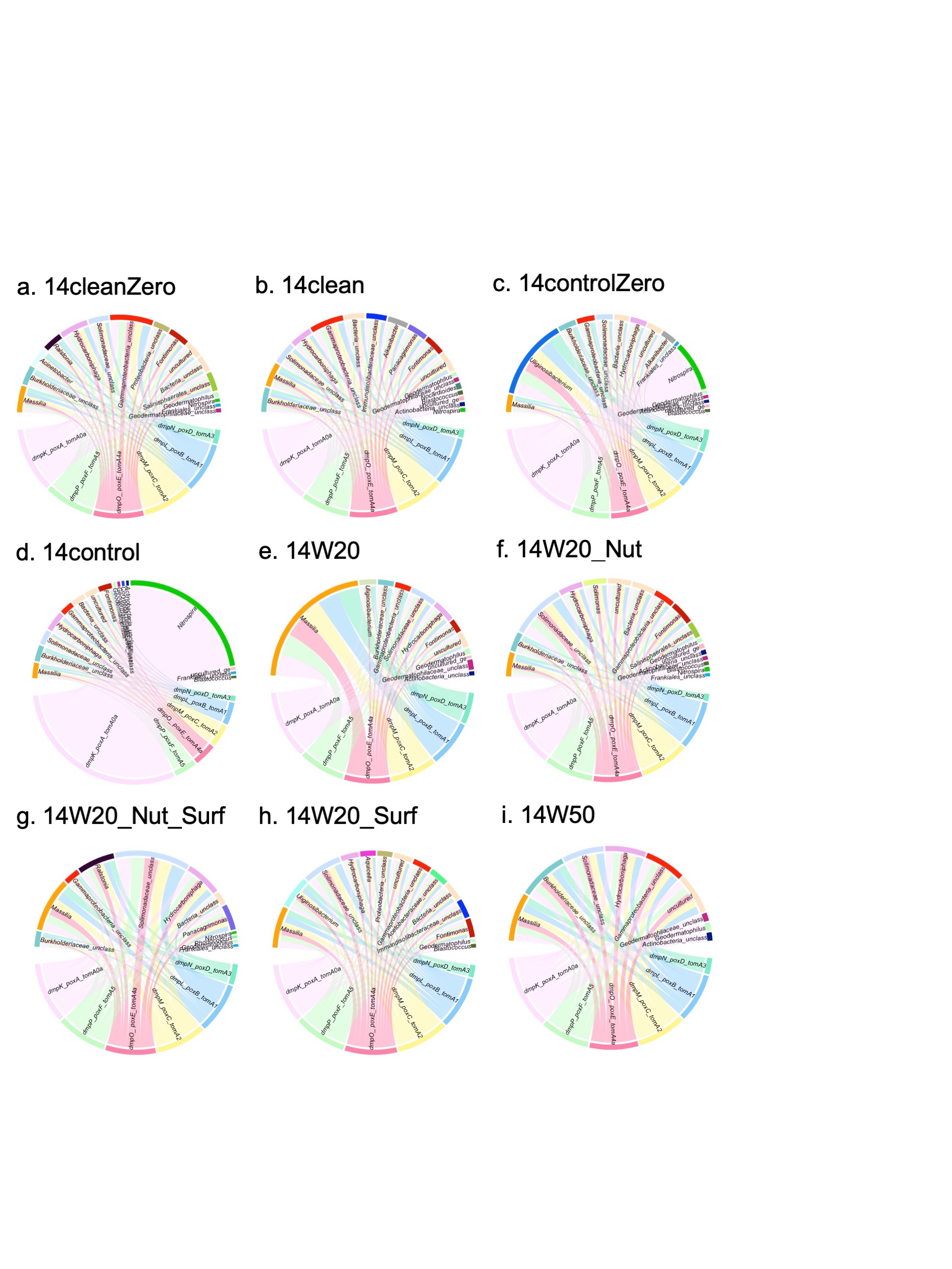


**Fig. S11** Phylotypes associated with phenol/toluene monooxygenase genes in the 14cleanZero(a), 14clean (b), 14controlZero (c), 14control (d), 14W20 (e), 14W20_Nut (f), 14W20_Nut_Surf (g), 14W20_Surf (h), 14W50 (i). To limit the number of phylotypes on each figure, the relative abundance threshold was set to > 0.005.
